# Developing a genetic manipulation system for the Antarctic archaeon, *Halorubrum lacusprofundi*: investigating acetamidase gene function

**DOI:** 10.1038/srep34639

**Published:** 2016-10-06

**Authors:** Y. Liao, T. J. Williams, J. C. Walsh, M. Ji, A. Poljak, P. M. G. Curmi, I. G. Duggin, R. Cavicchioli

**Affiliations:** 1 School of Biotechnology and Biomolecular Sciences, The University of New South Wales, Sydney, New South Wales, 2052, Australia; 2 School of Physics, The University of New South Wales, Sydney, New South Wales, 2052, Australia; 3 The ithree institute, University of Technology Sydney, Broadway, New South Wales, 2007, Australia; 4 Bioanalytical Mass Spectrometry Facility, The University of New South Wales, Sydney, New South Wales, Australia

## Abstract

No systems have been reported for genetic manipulation of cold-adapted *Archaea*. *Halorubrum lacusprofundi* is an important member of Deep Lake, Antarctica (~10% of the population), and is amendable to laboratory cultivation. Here we report the development of a shuttle-vector and targeted gene-knockout system for this species. To investigate the function of acetamidase/formamidase genes, a class of genes not experimentally studied in *Archaea*, the acetamidase gene, *amd3*, was disrupted. The wild-type grew on acetamide as a sole source of carbon and nitrogen, but the mutant did not. Acetamidase/formamidase genes were found to form three distinct clades within a broad distribution of *Archaea* and *Bacteria*. Genes were present within lineages characterized by aerobic growth in low nutrient environments (e.g. haloarchaea, *Starkeya*) but absent from lineages containing anaerobes or facultative anaerobes (e.g. methanogens, *Epsilonproteobacteria*) or parasites of animals and plants (e.g. *Chlamydiae*). While acetamide is not a well characterized natural substrate, the build-up of plastic pollutants in the environment provides a potential source of introduced acetamide. In view of the extent and pattern of distribution of acetamidase/formamidase sequences within *Archaea* and *Bacteria*, we speculate that acetamide from plastics may promote the selection of *amd/fmd* genes in an increasing number of environmental microorganisms.

The coldest lake known to support microbial growth is Deep Lake in Antarctica where temperatures drop to −20 °C^1^. Liquid water remains at these temperatures because the lake is hypersaline (~10× marine salinity). It is a closed, isolated marine-derived system that separated from the Southern Ocean ~3,500 years ago^2^. Genomic, metagenomic and metaproteomic studies have revealed that the lake community has a number of remarkable features: a low complexity community of haloarchaea that support a high level of community wide, intergenera gene exchange^2^; genome variation and niche adaptation occurring at the level of genera and strains^2^,^3^,^4^; virus-host interactions involving invasion, evasion and adaptation strategies^5^. The three most abundant members that represent ~72% of the entire lake community have been cultivated and their genome sequences determined: *Halohasta litchfieldiae* (~44%), DL31 (an undescribed genus; ~18%) and *Halorubrum lacusprofundi* (~10%)^2^. An additional species which represents a minor fraction of the lake community has also been isolated and sequenced: DL1 (*Halobacterium* sp.; ~0.3%)^2^. By being able to cultivate the abundant members (representing about three-quarters of the lake’s cellular population), the Deep Lake system is unusual compared to most environmental systems where typically <1% can be isolated and grown as axenic cultures in the laboratory^6^.

*Hrr. lacusprofundi* is the most readily isolated species, typically representing the majority of isolates forming colonies on plates from Deep Lake, and was the first psychrophilic member of the *Archaea* formally described^7^,^8^. In addition to the studies described above, *Hrr. lacusprofundi* has been the subject of genomic^9^,^10^,^11^,^12^,^13^, protein^14^,^15^,^16^, membrane lipid^17^, and physiological^18^,^19^,^20^ studies.

To date, *Methanococcoides burtonii* (isolated from Ace Lake in the same region of Antarctica)^21^ has served as the main model for examining cold adaptation of *Archaea*^22^,^23^. However, *M. burtonii* is not readily cultivated on plates and a genetic system for it has not been developed. The development of genetic manipulation systems for *Archaea* has greatly facilitated understanding of their molecular biology^8^,^24^. Transformation systems for haloarchaea were developed in the late 1980s^25^, largely built around the well-studied species, *Haloferax volcanii* and *Halobacterium salinarum*^26^,^27^,^28^,^29^, leading to the development of molecular genetic tools including selectable markers^27^,^30^,^31^,^32^,^33^, shuttle vectors^30^,^34^,^35^,^36^,^37^,^38^,^39^,^40^, reporter constructs^41^,^42^,^43^,^44^, overexpression systems^40^, and gene knockout systems^28^,^29^,^31^,^32^.

Antibiotics and resistance genes for *Archaea* are different to those for *Bacteria* but can have parallels with those from *Eucarya*. Mevinolin, which is derived from the fungus *Aspergillus*, inhibits 3-hydroxy-3-methylglutaryl coenzyme A reductase (HmgA), which is an essential enzyme in the synthesis of isoprenoid lipids in *Archaea*. An overexpression mutant of this gene from *Hfx. volcanii* provides resistance to mevinolin^38^,^45^. In humans, cholesterol is produced from the mevalonate pathway and statins that target HmgA are used for controlling cholesterol levels. In haloarchaea, overexpression of the gene that encodes HmgA (*hmgA*) can also provide resistance to the statins fluvastatin, simvastatin and pravastatin^33^.

In this study we aimed to develop a system for genetic manipulation of *Hrr. lacusprofundi* ACAM34. We targeted an acetamidase/formamidase (*amd/fmd*) gene because they have not been experimentally characterized in *Archaea*. *Hrr. lacusprofundi* encodes three *amd/fmd* genes sharing 29–42% identity, and here we define them as *amd1* (Hlac_1866), *amd2* (Hlac_2016) and *amd3* (Hlac_2285). In recent proteomic studies of *Hrr. lacusprofundi* ACAM34, *amd3* was identified as an abundant protein under a variety of growth conditions (Liao Y and Cavicchioli R, unpublished results). Amd/Fmd enzymes catalyze a single-step reaction (hydrolysis of acetamide or formamide) for which substrate (acetamide or formamide) is readily commercially available. We reasoned that a gene knockout would be unlikely to be lethal as the enzyme does not function in central metabolism, and the gene appears to be mono-cistronic, thereby reducing the likelihood of gene inactivation generating polar effects. Our study describes the development of transformation, construction of a shuttle-vector, disruption and phenotypic characterization of an *amd3* mutant, and discusses the ecological and evolutionary significance of the findings.

## Results

### Plasmid construction

**Figure 1:**
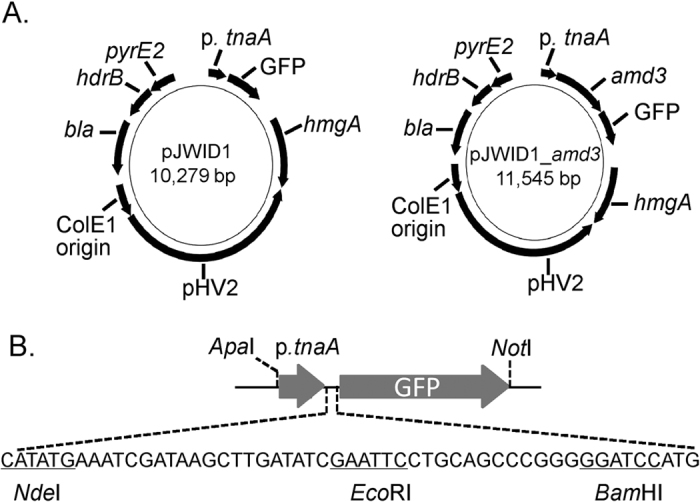
Plasmid maps of pJWID1 and pJWID1_*amd3*. (**A**) The *E. coli*-haloarchaea shuttle plasmid pJWID1 is based on pIDJL40^39^,^40^ which contains the SmRS-GFP gene under the control of the tryptophan-regulated *p.tnaA* promoter, and the selection markers *bla* (ampicillin resistance in *E. coli*), *pyrE2* (for selection in pyrimidine auxotrophs) and *hdrB* (thymidine auxotrophy in rich media). pJWID1 has the additional selection marker, *hmgA* (resistance to statins including pravastatin). In pJWID1_*amd3* the *amd3* gene was inserted at the *Nde*I and *Eco*RI sites of pJWID1. (**B**) Expanded view of the cloning sites in the region containing the *p.tnaA* promoter and GFP gene.

The *Hfx. volcanii*-*Escherichia coli* shuttle vector pIDJL40^39^,^40^ encodes the ColE1 origin of replication and *bla* gene for ampicillin selection in *E. coli*, and the *Hfx. volcanii* pHV2 origin of replication and *pyrE2* for selection in pyrimidine auxotrophs and *hdrB* gene for selection using thymidine auxotrophy in rich media. It also harbors a soluble-modified red-shifted green fluorescent protein (smRS-GFP) under the control of the tryptophan-inducible promoter from the *tnaA* gene of *Hfx. volcanii* (*p.tnaA*) that is flanked by the *Hfx. volcanii* L11e ribosomal protein gene terminator (t.L11e) and a synthetic terminator (t.Syn) comprising a T track flanked by G/C-rich sequences. In order to construct a plasmid that conferred resistance to statin drugs, pJWID1 was constructed by cloning the up-regulated mutant of the *hmgA* gene from *Hfx. volcanii*^33^ into pIDJL40 (see Methods and Fig. 1). Strains and plasmids used in this study are listed in Table 1, and PCR primers in Table S1.

### Development of DNA transformation protocol for *Hrr. lacusprofundi*

In the initial testing phases for developing transformation, minimal inhibitory concentrations of novobiocin (0.05 μg mL^−1^), mevinolin (0.01 μg mL^−1^), simvastatin (0.005 μg mL^−1^), fluvastatin (0.05 μg mL^−1^) and pravastatin (1 μg mL^−1^) were determined and competent cells prepared using a PEG-based procedure^26^. Initially, the shuttle vectors pJAM202^35^ and pCBD-sec11b^37^ were tested, which confer novobiocin resistance from the *Haloferax* strain Aa2.2 *gyrB* gene^34^, but transformants of *Hrr. lacusprofundi* were not obtained. Success was achieved using PEG-mediated transformation and selection of pJWID1 using 2.5 μg mL^−1^ pravastatin, with clear differences in resistance observed between transformed (up to 20 μg mL^−1^) and untransformed cells (Fig. S1). While the *hmgA* gene can confer resistance to mevinolin, simvastatin and fluvastatin in *Hfx. volcanii*^33^, effective resistance was only observed for pravastatin in *Hrr. lacusprofundi*.

The plasmid was prepared in an *E. coli dam dcm* strain because plasmid methylation was reported to significantly reduce transformation efficiency in *Hfx. volcanii*^46^. Transformation efficiency of *Hrr. lacusprofundi* increased from 1 ng to 1 μg of DNA with the highest efficiency of ~9 × 10^7^ transformants per μg obtained using 1 μg of pJWID1 (Fig. S2), an efficiency that is similar to transformation of *Haloferax* strain Aa 2.2 with the novobiocin resistance plasmid, pHK2^30^. Using 10 μg of DNA, the total number of transformants was similar to using 1 μg, translating to ~10-fold decrease in efficiency per μg (Fig. S2). The data indicate there is no benefit to using more than 1 μg of intact plasmid DNA for transforming *Hrr. lacusprofundi*. Pravastatin resistant colonies were not observed if plasmid DNA was omitted, and the transformation procedure which uses EDTA and PEG_600_ did not reduce cell viability (data not shown). Plasmid stability was tested by growing transformed cells in liquid medium without antibiotic, plating cells on solid medium in the absence of antibiotic, and assessing the ability of the cells to grow on pravastatin (2.5 μg mL^−1^) containing plates (Fig. S3). All colonies tested (total 50) were sensitive, indicating the plasmid was readily cured. The relative ease of curing provides potential benefit for experiments requiring plasmid loss. The plasmid was also effectively maintained in strains in the presence of pravastatin (2.5 μg mL^−1^).

### GFP expression from pJWID1

**Figure 2:**
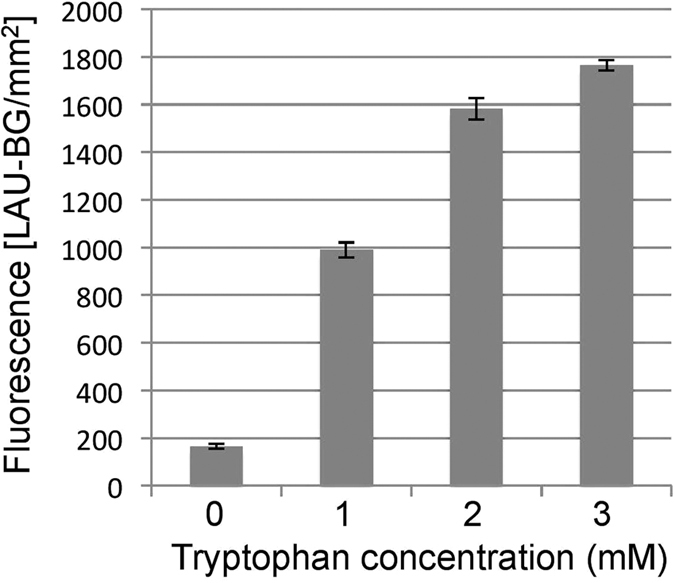
Tryptophan induction of GFP expression from pJWID1 in *Hrr. lacusprofundi*. Quantitative measurement of fluorescence emission in response to 0, 1, 2 and 3 mM tryptophan induction. GFP expression levels of *Hrr. lacusprofundi* harboring plasmid pJWID1 increased with tryptophan concentration. The fluorescence is given in light absorbance units per mm^2^ [(LAU-BG)/mm^2^]. Error bars represent standard error of three replicate cultures.

The smRS-GFP gene is under the control of the *p.tnaA* promoter but the coding sequence is out of frame with the expected start codon (within *Nde*I) in *Hfx. volcanii* (Fig. 1)^39^. The fact that GFP expression occurs (Fig. 2, Fig. S4) demonstrates that the translation machinery in *Hrr. lacusprofundi* is able to recognize and initiate translation of the GFP ORF in pJWID1. Moreover, expression levels increased with tryptophan concentration (1–3 mM) demonstrating that tryptophan induction also functioned effectively in *Hrr. lacusprofundi*. This pattern of expression occurred throughout the growth phase from mid-log to mid-stationary phase (data not shown). Expression of GFP was sufficient to readily enable fluorescence microscopy observation of cells (Fig. S4) and quantification of GFP using a fluorescence scanner (Fig. 2). The ability to detect GFP fluorescence in *Hrr. lacusprofundi* provides the potential for constructing reporter-fusions, tracking plasmid transfer, and performing flow activated cell sorting and GFP-fusion, protein localization experiments (also see **Plasmid expression of** ***amd3*** below).

### Construction of gene knockouts using plasmid-mediated, gene inactivation

The *hmgA* gene conferring pravastatin resistance was the only effective antibiotic selection marker we identified (see **Development of DNA transformation protocol for** ***Hrr. lacusprofundi***above). To construct a gene knockout, we initially considered developing a pop-in, pop-out approach that uses *pyrE* auxotrophs^32^. *Hrr. lacusprofundi* possesses one orotate phosphoribosyltransferase, *pyrE* gene (Hlac_0584). However, spontaneous *pyrE* mutants were not isolated following the passaging of cells through increasing concentrations (200–500 μg mL^−1^) of 5-fluoroorotic acid (1–2.5-fold above the minimum inhibitory concentration) in the presence of uracil (50 μg mL^−1^). The approach was therefore abandoned in favor of a strategy that used a suicide plasmid^32^ and gene exchange with an *hmgA* inactivated *amd3* gene.

**Figure 3:**
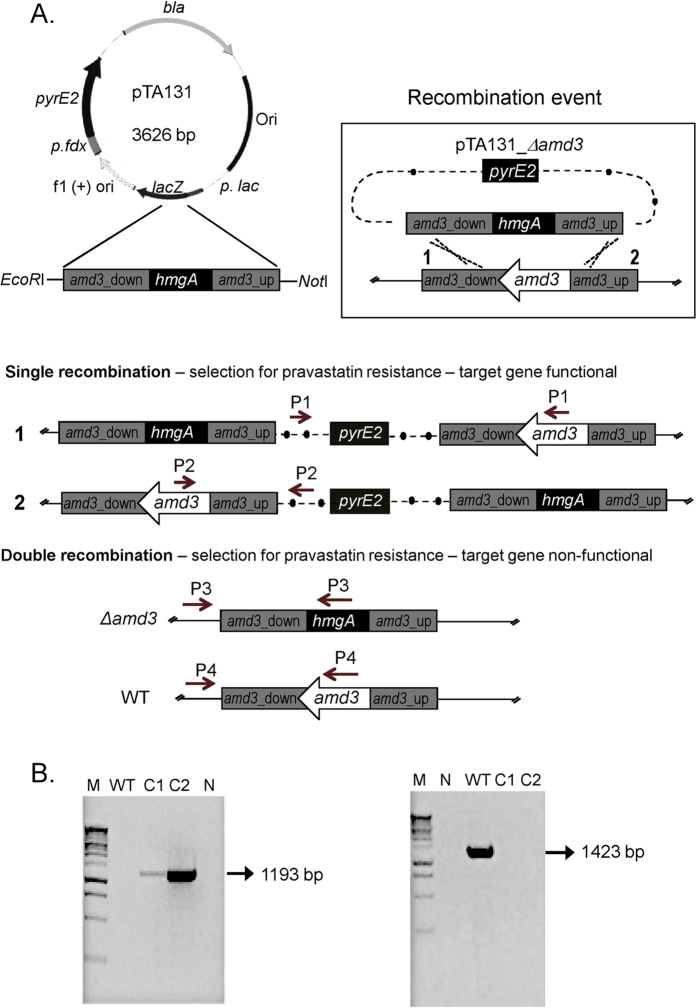
Construction of *amd3* gene disruption. (**A**) Pravastatin resistant cells arising from the transformation of pTA131_Δ*amd3* into wild-type *Hrr. lacusprofundi* can occur due to a single (plasmid insertion) or double (gene replacement) recombination event. Only the double recombination event produces a defective *amd3* gene. Four groups of primers (P1, P2, P3 and P4) were used to determine if single or double recombination events occurred. The region denoted *amd3*_down corresponds to a 950 bp fragment containing the last 214 bp of *amd3* and 736 bp of the downstream sequence, and the region denoted *amd3*_up corresponds to a 970 bp DNA fragment containing 225 bp of the 5′ coding region of *amd3* and 745 bp of the upstream sequence (see Methods). In the section showing double recombination, the gene structure and diagnostic primers are shown for the *amd3* disruption (Δ*amd3*) and wild-type strain (WT). (**B**) A PCR product (1193 bp; left gel image) using the P3 primers was diagnostic of a double recombination event, whereas a PCR product (1423 bp; right gel image) using P4 primers was diagnostic of the wild-type genes being present. (**A,B**) Lane M, 1 kb DNA ladder; Lane N, no DNA (negative control); Lane WT, untransformed *Hrr. lacusprofundi*; Lane C1–C2, two strains with double recombination events. C2 was chosen as the *Hrr. lacusprofundi* Δ*amd3* strain. The results of other primer sets for C1 and C2 are show in Fig. S5.

To inactivate the *amd3* gene, plasmid pTA131_Δ*amd3* was constructed by cloning the *amd3* gene that was inactivated by the insertion of the *hmgA* gene, into pTA131^31^(see Methods). Pravastatin resistant (2.5 μg mL^−1^) colonies of *Hrr. lacusprofundi* arising from transformation of pTA131_Δ*amd3* can arise from a single recombination event leading to plasmid integration, or a double recombination event leading to exchange of the wild-type gene for the disrupted gene (Fig. 3A). Genomic DNA extracted from 10 pravastatin resistant colonies was screened by PCR to discriminate between single and double recombination events (see Methods). Two transformants gave a single band using P3 primers, and no product using P1, P2 and P4 primers (Fig. 3B, Fig. S5), which was diagnostic for a double recombination event. The single band (Fig. 3B) matched the size of the product expected for P3 primers (1193 bp), and analysis of the DNA sequence of the PCR product for each of the two transformants confirmed the presence of the *hmgA* gene within *amd3*. One of the two strains was designated *Hrr. lacusprofundi* Δ*amd3.* The frequency of achieving double recombination (2/10 clones) is similar to that previously achieved in *Hfx. volcanii* for the construction of a *pyrE* gene disruption (6/16 clones)^32^.

### Assessment of the phenotype conferred by *amd3*

**Figure 4:**
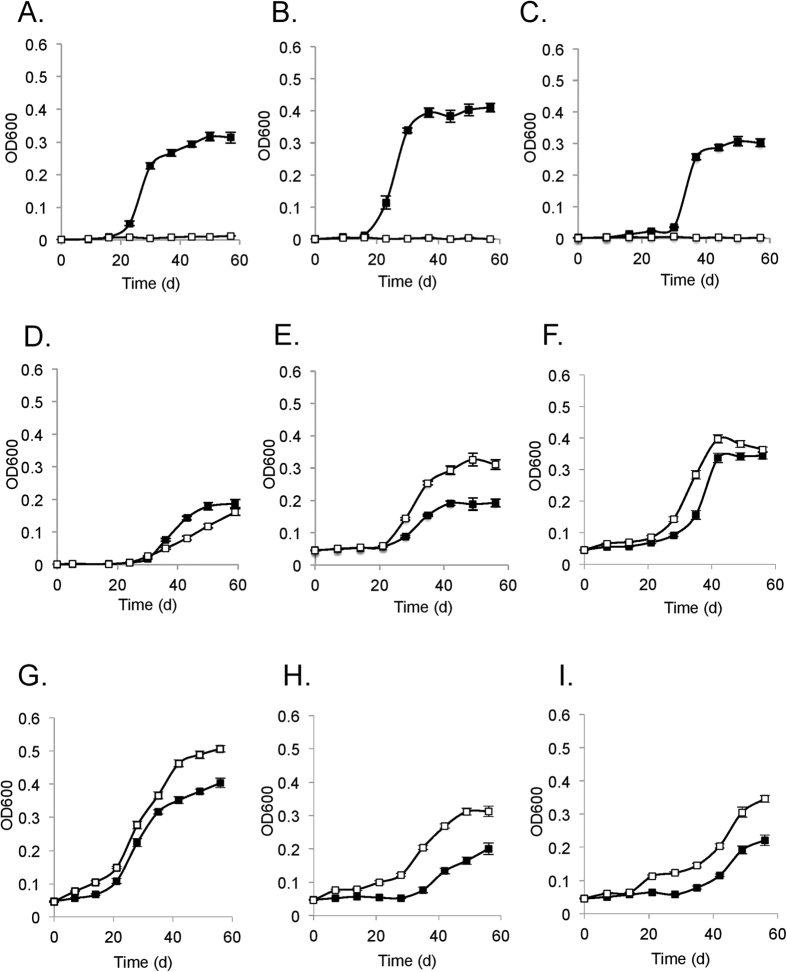
Phenotypic characterization of *Hrr. lacusprofundi* ACAM34 wild-type and Δ*amd3* mutant strains. Growth of wild-type (full symbols) and Δ*amd3* mutant (open symbols) on (**A**) 10 mM pyruvate and 10 mM acetamide; (**B**) 5 mM ammonium and 10 mM acetamide; (**C**) 10 mM acetamide; (**D**) 10 mM pyruvate and 10 mM formamide; (**E**) 10 mM pyruvate and 10 mM nicotinamide; (**F**) 10 mM pyruvate and 10 mM glutamine; (**G**) 10 mM pyruvate and 10 mM asparagine; (**H**) 5 mM ammonium and 10 mM glutamine; (**I**) 10 mM glutamine. Error bars represent standard error of three replicate cultures.

To assess the function of *amd3*, the wild-type and mutant were grown in media containing various amide substrates (acetamide, formamide, glutamine, asparagine, nicotinamide, urea) and growth assessed using these compounds as a sole carbon, nitrogen, or carbon and nitrogen source in defined media (Fig. 4). The wild-type grew using 10 mM acetamide as a sole source of nitrogen (with 10 mM pyruvate as the carbon source; Fig. 4A), sole source of carbon (with 5 mM ammonium as the nitrogen source; Fig. 4B), or as the sole source of both carbon and nitrogen (10 mM acetamide only; Fig. 4C). In contrast, under the same conditions the mutant was unable to grow (Fig. 4A–C). The phenotypic distinctions between wild-type and mutant were marked, and the results indicate that *amd3* is a functional acetamidase gene that enables *Hrr. lacusprofundi* to grow on acetamide.

Growth using 10 mM formamide demonstrated formamide could be used by *Hrr. lacusprofundi* as a sole source of nitrogen (Fig. 4D), but not as a sole source of carbon (data not shown). The Δ*amd3* mutant was also capable of growth with formamide as a sole source of nitrogen (Fig. 4D). The growth of the mutant lagged behind the wild-type indicating that Amd3 had activity on formamide but *Hrr. lacusprofundi* possessed other amidases (possibly Amd1 and/or Amd2) that also functioned as a formamidase to enable cells to grow.

Although urea did not support growth of the wild-type or mutant (data not shown), nicotinamide, glutamine or asparagine did support growth as a sole source of nitrogen (Fig. 4E–G). *Hrr. lacusprofundi* was not capable of growth using nicotinamide or asparagine as a sole source of carbon (data not shown), but both the wild-type and mutant did grow with glutamine as a sole source of carbon, or as a sole source of both carbon and nitrogen (Fig. 4H,I), albeit more slowly than growth on acetamide (Fig. 4C). However, unlike growth on formamide, for nicotinamide, glutamine or asparagine, the growth of the mutant was superior to the wild-type, indicating disruption of *amd3* had pleiotropic effects. It is not apparent why inactivation of *amd3* would lead to better growth on these substrates. This could possibly occur if Amd3 (in the wild-type strain) produces a metabolite that negatively effects (*e.g.* allosteric) the regulation of gene expression or activity of the enzymes involved in catabolizing nicotinamide, glutamine, and asparagine.

### Plasmid expression of *amd3*

**Figure 5:**
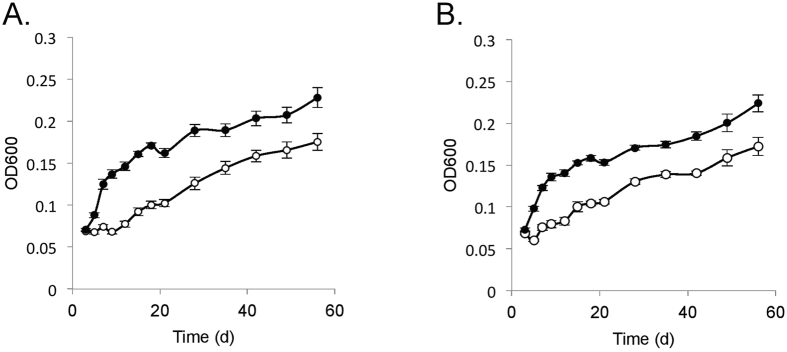
Growth of *Hrr. lacusprofundi* wild-type harboring pJWID1 or pJWID1_*amd3*. Growth of *Hrr. lacusprofundi* harboring pJWID1 (open symbols) or pJWID1_*amd3* (closed symbols) with (**A**) acetamide as the sole source of carbon; (**B**) acetamide as the sole source of carbon and nitrogen. Error bars represent the standard error of three replicate cultures.

The *amd3* gene was cloned in pJWID1 to construct pJWID1_*amd3* (Fig. 1). The *amd3* gene was cloned from start codon to stop codon downstream of the *p.tnaA* promoter with the open reading frame terminating prior to the beginning of the GFP open reading frame (i.e. not a translational fusion). Tryptophan (3 mM) was used to induce *p.tnaA*-mediated *amd3* expression in *Hrr. lacusprofundi*. Tryptophan could not support growth as carbon (with 5 mM ammonium as nitrogen), nitrogen (with 10 mM pyruvate as carbon) or sole source of carbon and nitrogen (data not shown). GFP was expressed from this plasmid although fluorescence levels were lower than for pJWID1 (Fig. S6). Background fluorescence in the absence of tryptophan was also observed for both pJWID1 and pJWID1_*amd3*, although again it was lower for pJWID1_*amd3* (Fig. S6). The data indicate that in the absence of tryptophan, expression from *p.tnaA* is not completely repressed in *Hrr. lacusprofundi* (i.e. somewhat leaky expression), and GFP expression can co-occur with expression of cloned genes in pJWID1. To assess the effects of increased gene copies and/or gene expression of *amd3* on the ability to utilize acetamide, growth was compared between wild-type *Hrr. lacusprofundi* harbouring either pJWID1 or pJWID1_*amd3* (Fig. 5). Enhanced growth was observed with pJWID1_*amd3*, particularly when acetamide was the sole carbon or sole carbon and nitrogen source (Fig. 5).

### Acetamidase enzyme activity

Acetamidase enzyme activity was determined (in triplicate) for the wild-type and Δ*amd3* mutant grown in media containing acetamide plus pyruvate and ammonium (to support growth of the mutant), and wild-type cells harbouring pJWID1 or pJWID1_*amd3* grown with acetamide as the sole source of carbon and nitrogen. Activity for the Δ*amd3* mutant was very low (0.04 ± 0.01 U mg^−1^) compared to the wild-type (18.1 ± 0.4 U mg^−1^), and somewhat higher for cells harbouring pJWID1_*amd3* (28.6 ± 0.5 U mg^−1^) compared to pJWID1 (23.8 ± 0.1 U mg^−1^). These enzyme activity data are consistent with the inability of the Δ*amd3* mutant to utilize acetamide for growth (Fig. 4A–C), and pJWID1_*amd3* enhancing the ability of the wild-type strain to grow on acetamide (Fig. 5).

### Characterization of Amd/Fmd sequences in *Archaea* and *Bacteria*

To identify Amd/Fmd sequences, UniProtKB was searched using a word search (see Methods). To validate the approach, randomly sampled sequences from the word search were used in a BLAST search of UniprotKB and cross checked to the original set. From a total of 2500 BLAST matches, only two new sequences were obtained, illustrating the word search was effective at retrieving Amd/Fmd sequences. Sequences were clustered using a 90% identity cutoff (OTU0.9) with clusters containing between one and 291 sequences. The 1323 OTU0.9 manually curated clusters consisted of 139 from *Archaea* and 1184 from *Bacteria* (Table S2). For *Archaea*, sequence diversity was highest in *Halobacteria* (haloarchaea) with 94 clusters and *Thermoprotei* (14 clusters), and for *Bacteria*, sequence diversity was highest in *Actinobacteria* (329), *Alphaproteobacteria* (245) and *Bacilli* (172) (Fig. S7).

**Figure 6:**
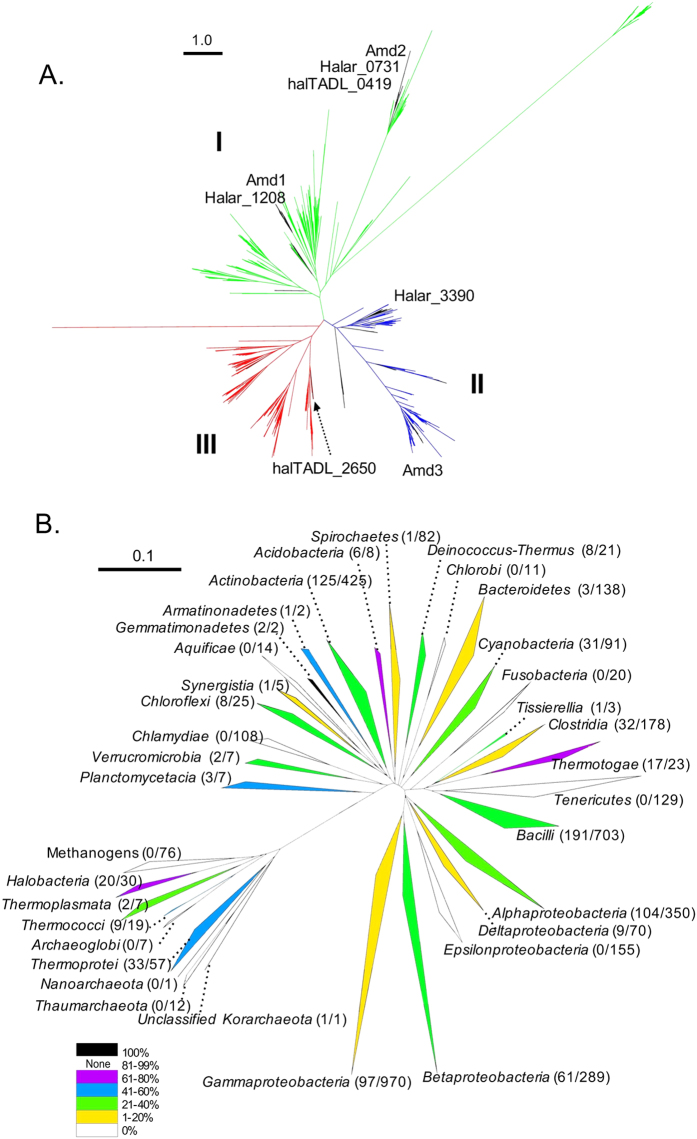
Diversity distribution and abundance of Amd/Fmd sequences within *Archaea* and *Bacteria*. (**A**) Maximum likelihood tree of Amd/Fmd protein sequence clusters (OTU0.9) showing three distinct clades (Clade 1, green; Clade II, blue; Clade III, red) with the root of the tree (bootstrap value, 0.996) being of bacterial origin. Clusters of archaeal sequences are highlighted (black branches). The Deep Lake haloarchaeal Amd/Fmd sequences (*Hrr. lacusprofundi*: Amd1, Amd2, Amd3; undescribed genus DL31: Halar_0730, Halar_1208, Halar_3390; *Hht. litchfeldiae*: halTADL_0419, halTADL_2650) were distributed in Clade I (Amd1, Halar_1208, Amd2, Halar_0731 and haltADL_0419), Clade II (Amd3 and Halar_3390) and Clade III (haltADL_2650). Scale bar represents 1 amino acid variation per aligned position. (**B**) Neighbour-joining tree of 16S rRNA gene sequences for closed genomes from *Archaea* and *Bacteria* which possess Amd/Fmd sequences. 16S rRNA gene sequences represented at class or phylum level. Clades are colored according to the proportion of closed genomes that contain Amd/Fmd sequences. The number of Amd/Fmd sequences relative to total number of closed genomes in a clade is shown in parentheses after the name of the clade. Bacterial clades with less than 10 sequences that do not contain Amd/Fmd sequences are not displayed. The scale bar represents 0.1 changes per base position.

Phylogenetic analyses were performed using one sequence from each cluster. Three distinct Amd/Fmd clades were apparent in trees constructed using all clusters (Fig. 6A, Fig. S8), or a subsample of equal numbers of archaeal and bacterial sequences (Fig. S9). The clade structure was robust (1000 bootstraps) and the three clades rooted (bootstrap 0.996) to a bacterial origin with archaeal sequence ‘islands’ distributed within bacterial clusters. Archaeal sequences were present in each clade, although Clade III consisted almost exclusively of bacterial sequences. Sequences from individual organisms tended to be distributed amongst the clades rather than being confined to a single clade. For the Antarctic haloarchaea, Clade I contained Amd1, Halar_1208, Amd2, Halar_0731 and haltADL_0419; Clade II, Amd3 and Halar_3390; Clade III, haltADL_2650. Amd3 clustered with sequences from an uncultured archaeon and two other haloarchaea (*Natronomonas pharaonis* and *Halopiger xanaduensis*) and more distantly to sequences from *Clostridia* and *Bacilli* (Fig. S8). Amd1 and Amd2 do not have high sequence identity (42%) and were in subclades of Clade I. The *Hht. litchfeldiae* Amd/Fmd sequence halTADL_2650 was one of only three archaeal sequences in Clade III, and clustered with sequences from *Haloquadratum walsbyi* and an unclassified bacterium YEK0303, and more distantly with a *Bacillus* species (Fig. S8).

Closed genomes were used to quantify the representation (including presence/absence) of Amd/Fmd sequences within taxa (Table S3; Fig. 6B). From 215 archaeal genomes, 65 contained Amd/Fmd sequences at an average 1.28 per genome, with 703 for *Bacteria* at an average 1.40 per genome (Fig. S10). For *Archaea*, 20 of 30 closed genomes from haloarchaea contained Amd/Fmd sequences, and for those carrying the genes they had the highest average number (1.75) of genes per genome for *Archaea* (Fig. S10). The archaeal species which contained the largest number of Amd/Fmd sequences was *Haloarcula marismortui* which contained four (Table S4). The genomes of *Archaeoglobi*, *Thaumararchaeota* *Nanoarchaeota* and all the methanogens (*Methanobacteria*, *Methanococci*, *Methanomicrobia*, *Methanopyri*) did not contain Amd/Fmd sequences (Table S4; Fig. 6B). For *Bacteria*, Amd/Fmd sequences were present in 402 species representing 225 genera (Table S4). The *Bacteria* that tended to have Amd/Fmd sequences (≥70% of the taxa) were *Gemmatimonadetes*, *Acidobacteria* and *Thermotogae* (Fig. 6B). *Starkeya*, *Modestobacter*, *Bradyrhizobium*, *Agromonas* and *Opitutaceae* sp. TAV5 contained ≥5 Amd/Fmd sequences per genome, with the largest number (8) in *Starkeya novella* (Table S4). Bacterial genomes that lacked Amd/Fmd sequences were from *Epsilonproteobacteria*, *Tenericutes*, *Chlamydiae*, *Fusobacteria*, *Aquificae* and *Chlorobi* (Fig. 6B, Table S2).

### Concentration of acetamide in aquatic samples

In order to assess the likelihood of acetamide serving as an environmental growth substrate, the acetamide concentration was determined in water samples from Deep Lake, hypersaline lakes from the Rauer Islands and the Southern Ocean (see Methods). Acetamide was detected at levels similar to the background levels present in Milli-Q water. The presence of 1–2.5 μM acetamide in the controls probably derives from the high-density polyethylene tubes the water samples were stored in. When the standard curve was corrected for background acetamide (Fig. S11), only two ocean samples were above control levels (highest 4.74 μM).

## Discussion

Here we report the first procedure for performing gene transfer and gene knockouts for a psychrophilic member of the *Archaea*. Even for psychrophilic bacteria, few systems are available for genetic manipulation^47^,^48^,^49^,^50^,^51^,^52^. *Hrr. lacusprofundi* is an important member of the Deep Lake community, representing ~10% of the population throughout the depth of the lake. Because of the relative ease of isolation from environmental samples^3^, it was the first psychrophilic member of the *Archaea* formally described^7^,^8^. *Hrr. lacusprofundi* is capable of growth in the laboratory at 4 °C, and very slowly at 0 or −1 °C^7^,^18^. Intergenera gene exchange and population level genetic variation is a feature of the Deep Lake community^2^,^3^,^5^. The development of genetic manipulation of strain ACAM34 provides considerable scope for probing mechanisms of adaptation and gene exchange in this species.

### Physiological and ecological significance of acetamide

The ability of *Hrr. lacusprofundi* to utilize acetamide as a sole source of both carbon and nitrogen, but formamide only as a sole source of nitrogen, indicates that cells can utilize ammonium released from either source, but that only acetate and not formate can be used as a carbon source. This is consistent with genomic evidence for *Hrr. lacusprofundi*, which includes a capacity for acetate assimilation via the glyoxylate cycle^3^,^53^, but no evidence for known formate assimilation pathways. This is further corroborated by growth assessments which show that *Hrr. lacusprofundi* can utilize acetate as a sole source of carbon^3^,^7^. The enzyme activity data and pronounced phenotype of the *Hrr. lacusprofundi* Δ*amd3* strain and relatively fast growth of the wild-type strain on acetamide illustrates that Amd3 functions effectively in substrate conversion and is unlikely to limit ammonium and acetate utilization pathways. Deamidation of formamide may be calatalyzed by an Amd3 homolog; possibly Amd1 and/or Amd2. The *Hrr. lacusprofundi* genome encodes genes for a putative asparaginase (COG0252: Hlac_2272) and nicotinamidase (COG1335: Hlac_2101, Hlac_2473) for the deamidation of asparagine and nicotinamide, respectively. A gene for glutaminase is not apparent in the *Hrr. lacusprofundi* genome; but glutamine can be assimilated (via glutamate) using glutamate synthase (GOGAT). Ammonium liberated by amidases can be assimilated via the glutamine synthetase-GOGAT cycle, which is present in *Hrr. lacusprofundi*^3^.

The role of amides such as acetamide and formamide as sources of carbon and/or nitrogen has been examined in fungi^54^, algae^55^,^56^ and *Bacteria*, including species of *Pseudomonas* and *Burkholderia*^57^,^58^,^59^, *Alcaligenes*^60^, *Nocardia*^61^, and *Mycobacterium*^62^. In the marine environment amides have been described as a potential nutrient source, and speculated to be derived from photodegradation of dissolved organic matter, from atmospheric input, or as a byproduct of an unspecified degradative metabolic pathway^56^. Acetamide is known to be generated through the pyrolytic cleavage of N-acetylated biopolymers such as chitin^63^ and peptidoglycan^64^, and constitutes a dominant N-containing product from the fragmentation of soil organic matter, sewage sludge, and chitin-containing biomass^63^,^65^,^66^,^67^. Acetamide is also a byproduct of the catabolism of nitroimidazole antibiotics by bacteria, through the reductive cleavage of the imidazole ring^68^,^69^,^70^. In the Antarctic environment, acetamide would not be expected to be produced by thermal degradation (such as by fire or volcanic eruptions) of natural biopolymers. Acetamide may be generated endogenously within cells as a transient intermediate from the breakdown of natural imidazoles or other compounds. Our analyses found acetamide in water samples approximated relatively high (μM) background concentrations. If a pool of acetamide is not maintained in cells (and released during cell lysis), whatever acetamide becomes available in the environment is likely to be rapidly metabolized. In the ocean, dissolved organic carbon (e.g. glucose) and dissolved free amino acids are in the ~1–20 nM range and can have a high flux through the labile pool of nutrients^71^,^72^, so if acetamide is metabolized as actively, concentrations may not exceed nM levels.

### Evolution of the Amd/Fmd sequences in *Archaea* and *Bacteria*

Our study appears to be the first to examine the diversity and phylogeny of Amd/Fmd sequences in *Archaea* and *Bacteria*. The analyses revealed the presence of three distinct clades of Amd/Fmd sequences which are predicted to originate from *Bacteria* with subsequent dissemination to *Archaea* (Fig. 6A). Experimental data is available for very few representatives of the tree. Our data for Amd3 indicate that it effectively converts acetamide. The only other experimentally studied enzyme from Cluster II, Uniprot KB ID Q50228 from *Methylophilus methylotrophus*, has highest activity on formamide and relatively low activity on acetamide, propionamide, butyramide, and acrylamide^73^,^74^. The Cluster I enzyme, UniprotKB ID O25836 from *Helicobacter pylori*, was reported to only catalyze the conversion of formamide^75^. While limited, the experimental data (particularly for Cluster II) indicate that functional specialization (substrate preference for acetamide vs formamide) has occurred following lineage-specific gene acquisition.

The highest number of Amd/Fmd sequences occurs in *Bacteria*, including *Modestobacter multiseptatus*, *Pseudonocardia dioxanivorans*, *Agromonas oligotrophica*, *Bradyrhizobium* spp., *Opitutaceae sp.* and *Starkeya novella*. *Bradyrhizobium* spp., *A. oligotrophica*, *M. multiseptatus* and *S. novella* are soil bacteria^76^,^77^,^78^,^79^,^80^; *P. dioxanivorans* was isolated from industrial sludge^81^,^82^; and the putative methylotroph *Opitutaceae* sp. TAV5 was isolated from the hindgut of a wood-feeding termite^83^. Gene duplication and specialization may enhance the competitiveness of these bacterial taxa that are capable of growth by scavenging nutrients in low nutrient environments (especially soil), including growth on single-carbon compounds.

While Amd/Fmd sequence distribution is wide-spread in *Archaea* and *Bacteria*, specific taxonomic groups lack them. These tend to be anaerobic or facultative anaerobic microorganisms from aquatic or terrestrial environments (*Archaeoglobi*, methanogens, *Epsilonproteobacteria*, *Aquificae* and *Chlorobi*) or parasites of animals and plants (*Tenericutes*, *Chlamydiae* and *Fusobacteria*). The only aerobic group lacking Amd/Fmd sequences are *Thaumarchaeota* which possess an ability to generate energy via ammonia-oxidation^84^,^85^. The fact that methanogens lack these genes is notable for several reasons. Firstly, many methanogens are capable of growth on single-carbon compounds (CO_2_, methanol, formate). Secondly, acetamide is a potential source of acetate for acetoclastic methanogens. Thirdly, 76 methanogen genomes are available that represent species from diverse environments (e.g. deep-sea hydrothermal vents, Antarctic lakes, rice paddies, human and ruminant gastrointestinal tracts), and some are characterized as supporting a high level of horizontal gene transfer^86^,^87^,^88^. The lack of Amd/Fmd sequences in methanogens is suggestive of selection against retention of the genes rather than the existence of a barrier to acquiring the genes.

The Antarctic haloarchaea support a high level of intergenera gene exchange, including long stretches (up to 35kb) of identical DNA^2^. However, the three dominant genera have distinct metabolisms enabling them to utilize different lake substrates and providing selection for sympatric speciation^2^,^3^,^4^. The clustering of Amd1 with Halar_1208, and Amd2 and Halar_0731 with haltADL_0419 in Clade I may have arisen from intergenera transfer within the community in Deep Lake. However, the presence of Amd3 and Halar_3390 on distinct branches of Clade II, and halTADL_2650 in Clade III which contains very few archaeal sequences, suggests the genes arose from acquisition and selection for specific enzymatic properties as a means of fulfilling specific physiological function(s).

The pattern and extent of distribution of Amd/Fmd sequences within *Archaea* and *Bacteria* illustrates the evolutionary significance of the genes. The demonstration that acetamide can sustain microbial growth of *Hrr. lacusprofundi* as a sole source of carbon and nitrogen, is intriguing, and the sources and concentrations of acetamide in the Antarctic environment need to be accurately determined in order to consider the environmental cues controlling the selection of these genes. In industry, acetamide is used as a plasticizer and solvent^89^,^90^, and as a component of pesticides^91^, thereby providing avenues for acetamide to be introduced into the global environment as an industrial contaminant. Of great concern is the enormous build-up of environmental plastics^92^,^93^,^94^, as these potentially provide a significant anthropocentric source of acetamide. The selection of genetic variants with new capacities to utilize unnatural substrates has been documented for both atrazine pesticides and poly(ethylene terephthalate) plastics^95^,^96^,^97^. Recently, a *Rhodococcus* sp. was isolated which is capable of utilizing N,N-dimethylacetamide (DMAC) and its degradation product acetamide as sole sources of carbon and nitrogen^98^. DMAC is an acetamide-based compound that has become an important environmental pollutant that is widely used as an agrochemical and in a wide range of industries^98^. As the *amd/fmd* genes are already naturally occurring in a wide range of lineages, enhancing a community’s capacity to breakdown acetamide only requires dissemination and stable inheritance of one gene. As a result, we hypothesize that *amd/fmd* genes will arise in a greater number of microbial lineages and in a higher proportion of microbial communities that are increasingly exposed to introduced acetamide (*e.g.* plastics, pesticides, industrial waste).

## Methods

### Culture conditions, strains, plasmids and PCR primers

*Hrr. lacusprofundi* ACAM34 was grown in artificial Deep Lake vitamin succinate broth (ADLVSB)^7^ at 30 °C (see Supplementary information). The phenotype of wild-type and mutant strains was tested using various defined carbon and nitrogen substrates in modified DBCM2 medium^99^ which had yeast extract and peptone omitted. Acetamide, formamide, glutamine, asparagine, nicotinamide, and urea were used as amide substrates. Pyruvate was used a carbon source and ammonium as a nitrogen source. All carbon substrates were used a 10 mM, nitrogen substrates at 5 mM, and substrates used as both a source of carbon and nitrogen at 10 mM. *E. coli* strain c2925 (*dam, dcm*; New England Biolabs) was used to prepare unmethylated plasmid DNA for transformation of *Hrr. lacusprofundi*, and was grown in Luria-Bertani medium with 100 μg mL^−1^ ampicillin.

### Construction of shuttle vector pJWID1

Plasmid pJWID1 was constructed by cloning the up-regulated mutant of the *hmgA* gene from *Hfx. volcanii*^33^ into the *Hfx. volcanii*-*E. coli* shuttle vector pIDJL40^39^,^40^. Primers pJ_For and pJ_Rev were initially used to amplify the *hmgA* region from *Hfx. volcanii* DS2 and the product was used as template for a second PCR using the same reverse primer, and forward primer pJ_For_M that introduced two point mutations that up-regulate the promoter of *hmgA*. The resulting fragment was digested with *Pst*I and pIDJL40 was digested with *Nsi*I and alkaline phosphatase to create compatible ends for ligation, and ligation performed to generate plasmid pJWID1 (Fig. 1); the orientation and expected sequence of *hmgA* was verified by sequencing. A red fluorescence protein (mCherry) version of the plasmid (pJWID4) was also constructed (data not shown).

### Transformation, plasmid stability and GFP expression

*E. coli* was transformed using a standard heat-shock protocol^100^. For *Hrr. lacusprofundi*, a polyethylene glycol 600 (PEG_600_) procedure developed for *Hfx. volcanii*^101^ was used with modifications. Compositional changes were made to buffered salt water, regeneration and transformation dilution solutions (the full procedure is in Supplementary information). Cells were harvested for preparation of competent cells after growth in ADLVSB medium reached late-log phase: an optical density (OD_600_) of 0.8–1.0 (1.3–3.6 × 10^8^ cells mL^−1^). Transformants were selected on ADLVSB medium supplemented with 0, 0.05, 0.5, 1, 2.5, 5, 7.5, 10, 15 or 20 μg mL^−1^ of pravastatin (from 5 mg mL^−1^ stock dissolved in MilliQ-water). Plates were incubated in sealed plastic bags (with wet tissue to maintain moisture) at 30 °C for 15 d. To test transformation efficiency, cells were transformed with 1 ng, 10 ng, 100 ng, 1 μg or 10 μg of plasmid DNA. Plasmid stability was assessed by growing cells (inoculated 1:100) in liquid ADLVSB medium without antibiotic until late log phase, plating cells on solid medium in the absence of antibiotic, and testing the ability of 50 colonies to grow on solid medium containing 2.5 μg mL^−1^ pravastatin. To assess GFP expression, pJWID1 transformants were grown to mid-log phase in liquid ADLVSB medium supplemented with 2.5 μg mL^−1^ pravastatin, and 25 mL aliquots supplemented with 0, 1, 2 or 3 mM tryptophan. After further growth, cells were diluted with basal salts (3 M NaCl, 150 mM MgSO_4_, 40 mM KCl) as required to bring all cultures to OD _600_, 0.2. The fluorescence of samples (100 μL) in 96 well plates was quantified using a Fujifilm FLA-5000 Fluorescent Image Analyzer (Fujifilm, Tokyo, Japan) with a 473 nm excitation laser and Fujifilm LPB filter using Fujifilm Science Lab Image Gauge Ver 4.0 software. Basal salt solution was used as a blank and assessments were performed in triplicate, and standard error calculated. Cell fluorescence was also viewed and photographed with a digital microscope (Olympus BX61 microscopy with DP71 camera; Olympus, Tokyo, Japan) using bright-field or fluorescence-field imaging (Olympus WIBA filter).

### Construction of the *amd3* gene deletion strain

To construct pTA131_Δ*amd3*, an in-fusion high efficiency directional (HD) cloning system (Clontech/Takara Bio, Mountain View, CA, USA) was used for cloning multiple fragments in a single reaction. The plasmid pTA131^31^ was digested with *Eco*RI and *Not*I. The *hmgA* gene including promoter sequence was amplified by PCR from pJWID1 using primers *hmgA*_For and *hmgA*_Rev. A 970 bp DNA fragment containing the 225 bp of the 5′ coding region of *amd3* and 745 bp of the upstream sequence was PCR amplified. The primers used, Acet_up_For15 and Acet_up_Rev15_h, contain a 15 bp extension complementary to the *Not*I end of digested pTA131 and a 15 bp extension complementary to the 3′ end of *hmgA*, respectively. Similarly, a 950 bp fragment containing the last 214 bp of *amd3* and 736 bp of the downstream sequence was amplified by PCR with primers Acet_down_For15_h and Acet_down_Rev15, which contain a 15 bp extension complementary to the 5′ end of *hmgA* and a 15 bp extension complementary to the *Eco*RI end of digested pTA131. These three fragments and the linearized vector pTA131 (*Eco*RI/*Not*I digestion) were ligated simultaneously according to the manufacturer’s instructions. The pTA131_Δ*amd3* plasmid was transformed into competent *E. coli* c2925 and non-methylated plasmid DNA prepared. The plasmid pTA131_Δ*amd3* was transformed into *Hrr. lacusprofundi* and plated on ADLVSB medium containing 2.5 μg mL^−1^ pravastatin, and colonies screened by PCR using primers diagnostic for single or double recombination events (Table S1, Fig. 3B, Fig. S5).

### Plasmid expression of *amd3*

To control the expression of *amd3*, the gene was cloned to pJWID1 under the control of the *p.tnaA* promoter^39^ using the HD cloning system as described above. The *amd3* gene was amplified from *Hrr. lacusprofundi* genomic DNA using primers pJ_2285_FW and pJ_2285_RV, which contain 15 bp extensions complementary to *Nde*I and *Eco*RI ends of digested pJWID1. The fragment was ligated into linearized vector pJWID1 (*Nde*I/*Eco*RI) to generate pJWID1_*amd3*. The plasmid was sequenced to confirm the correct insertion event. The plasmid pJWID1_*amd3* (or pJWID1 as negative control) extracted from *E. coli* c2925 was transformed into *Hrr. lacusprofundi* Δ*amd3* and plated on ADLVSB medium supplemented with 40, 60, 80, 100, 120 or 150 μg mL^−1^ pravastatin. The plasmids were also transformed into the wild-type *Hrr. lacusprofundi* strain with selection on 2.5 μg mL^−1^ pravastatin.

### Acetamidase enzyme activity

*Hrr. lacusprofundi* harboring pJWID1 or pJWID1_*amd3* were inoculated 1:100 grown and grown for 40 d at 30 °C in 50 mL DBCM2 medium supplemented with 10 mM acetamide as the sole source of carbon and nitrogen plus 3 mM tryptophan to induce *p. tnaA*-mediated *amd3* expression. In addition, the wild-type and Δ*amd3* strain were inoculated 1:100 grown and grown for 40 d at 30 °C in 50 mL DBCM2 medium supplemented with 10 mM pyruvate, 1 mM ammonium and 10 mM acetamide, with the pyruvate and ammonium provided to support growth of the Δ*amd3* strain. Cells were pelleted by centrifugation for 20 min at 4,500 × *g*, washed three times in DBCM2 salt solution, and suspended in DBCM2 salt buffer supplemented with 2 mM EDTA (pH 7.2) and 0.4 mM phenylmethanesulphonylfluoride (PMSF). The suspensions were ultrasonically disrupted on ice using a Branson Sonifier 250 (Branson Ultrasonics, Danbury, CT) with the probe output set at 20% amplitude for five periods of 40 s (pulse cycle of 0.5 s on and 0.5 s off), with 40 s cooling on ice between periods of sonication to prevent excessive sample heating. The sonicate was centrifuged at 4,500 × *g* for 5 min to remove cell debris, and the cell free extract (supernatant) filtered through a 15 mL Amicon centrifugal concentration unit (Millipore, 25 Billerica, MA) with a 3 kDa cutoff by centrifugation at 5,000 × *g*, with three subsequent buffer exchanges with DBCM2 salt solution to remove EDTA and PMSF and the concentrate (~500 μL) stored at −80 °C until needed. Protein concentration was determined at 562 nm with a microplate reader using Thermo Scientific Pierce BCA Protein Assay Kit (Product No. 23225) according to manufacturer’s instructions. Acetamidase activity was determined by measuring the release of ammonium based on a phenol-hypochlorite ammonia detection protocol^102^. A standard reaction mixture (100 μL) containing 50 mM KH_2_PO_4_–K_2_HPO_4_ (pH 7.6), 150 mM NaCl, 10 mM acetamide and 100 μg of crude enzyme (cell free extract) was incubated at 30 °C for 1 h and the reaction terminated by the addition of 350 μL of reagent A (0.59 M phenol, 1 mM sodium nitroprusside). The color was developed by the addition of 100 μL of reagent B (2 M sodium hydroxide, 0.11 M sodium hypochlorite), with the mixture maintained at 30 °C in the dark for 20 min. Absorbance was measured at 600 nm with a microplate reader. The enzyme assays were performed in triplicate and a negative control that contained all reagents but no added cell free extract was included. Enzyme activity was calculated from a standard curve constructed using 0, 0.25, 0.5, 1, 2, 4, 6 and 8 mM NH_4_Cl. One unit of acetamidase activity was defined as the amount of enzyme that hydrolyzed acetamide to release 1 μM NH_3_ per minute under assay conditions.

### Phylogenetic analysis of Amd/Fmd sequences

Amd/Fmd protein sequences from *Archaea* and *Bacteria* were retrieved with a protein name search using “acetamidase”, “formamidase”, “Amd” and “Fmd” from UniProtKB database (18 March, 2016), and sequences recovered clustered with an identity cutoff of 90% (OTU0.9). Representative sequences of each cluster were interrogated and manually filtered to remove irrelevant sequences (e.g. transcriptional regulator of acetamidase genes). Six of the Deep Lake haloarchaeal Amd/Fmd sequences were in the search (*Hrr. lacusprofundi*: *amd1*, *amd2*, *amd3*; undescribed genus DL31: Halar_0730, Halar_1208, Halar_3390) but the two from *Hht. litchfeldiae* (halTADL_0419, halTADL_2650) were not and were manually added to the set and aligned against existing clusters using Clustal X 2.0^103^. Phylogenetic trees were constructed using Fasttree^104^ using the maximum likelihood method and the robustness of phylogeny tested using 1000 bootstraps. To test the ability of the word search to recover Amd/Fmd sequences, 50 sequences were randomly selected and BLAST used with each sequence against UniProtKB, and the top 50 hits from each BLAST search were cross-checked against the original datasets. To evaluate the effect of bias in the number of bacterial vs archaeal Amd/Fmd sequences, bacterial clusters were randomly subsampled to the same number of archaeal clusters, and the new dataset used for tree construction. To assess Amd/Fmd sequences in closed genomes, the 215 archaeal and 3872 bacterial genomes in Integrated Microbial Genomes (IMG) were searched (22 March, 2016) using the IMG Gene Cassette Search tool for the Pfam 03069 motif (includes acetamidase and formamidase). The 16S rRNA gene sequences corresponding to the archaeal and bacterial genomes were extracted from Silva non-redundant reference SSU database using genome names or retrieved manually from IMG. The 16S rRNA gene sequences were aligned using SINA aligner^105^ and classified with the least common ancestor method based on the different taxonomies hosted by SILVA. Common gaps in alignments were removed, and phylogenetic trees were constructed using the neighbor joining method in ARB^106^. The sequences were clustered according to their class or phylum 16S rRNA gene taxonomic classifications.

### Acetamide concentration of water samples

Water was collected in acid or ethanol washed, high-density polyethylene bottles from Deep Lake (12/2008; 11/2014; 12/2014), Rauer Islands lakes (01/2015) and the Southern Ocean (10/2008; 12/2008), and cryogenically stored at −80 °C. A total of 12 different samples from these systems, plus controls (100 μL each in duplicate) were dispensed into Pyrex screw cap glass culture tubes (Kimble, ThermoFisher, Sydney, Australia). An acetamide (Sigma, USA) standard curve was prepared in the 0–20 μg mL^−1^ range. To all samples, controls and standards (for standard curve), 5 μL (0.1 μg μL^−1^) of stable isotope labeled ^13^C_2_, ^15^N-acetamide internal standard (Medical Isotopes Inc., NH) was added. All samples to be analysed were dried in a vacuum centrifuge at ambient temperature (Savant speedvac, ThermoFisher, Sydney, Australia). To maximize recovery, over-drying was avoided and the hypersaline samples consisted of a moist slurry of crystals. Neat dichloromethane (1 mL) was added to each sample, control and standard, and shaken upright on a mixer platform (Intelli-Mixer, POCD Scientific, Sydney, Australia) for *~*2 h at ambient temperature. The liquid extract was transferred to clean glass culture tubes, taking care not to pick up salt crystals, and dried in a vacuum centrifuge (ambient temperature, *~*10 min). All samples were resuspended in 100 μL dichloromethane, vortexed briefly and transferred to GC vials with glass inserts. Stable isotope dilution GC/MS quantification of acetamide in lake water was carried out as described previously^107^ with specific modifications. Analyses were performed on a Hewlett-Packard 6890 plus gas chromatograph interfaced with an Agilent Technologies 5973 mass selective detector. A 4-mm-i.d. straight-walled silanized glass liner containing quartz wool was installed in the injection port and samples injected in the splitless mode (2 μL injection volume). Chromatography of underivatized acetamide was performed on a fused silica capillary column (free fatty acids column; Agilent J&W GC columns: HP-FFAP 50 m length, 0.2 mm id, 0.33 μm film thickness). Helium (BOC Gases, ultra-high purity) was used as the carrier gas at a flow rate of 1.4 mL min^−1^. The GC/MS conditions were as follows: injector temperature, 230 °C; transfer line, 230 °C; initial oven temperature, 40 °C (for 4 min); then increased to 190 °C at 5 °C min^−1^; then to 230 °C at 30 °C min^−1^, with a total run time of 45 min. Mass spectrometry analysis was performed using electron impact ionization mode, and conditions were as follows: electron energy, 70 eV; ion source temperature, 230 °C, MS Quad temperature 150 °C. Single ion monitoring was used to detect the molecular ions of acetamide (59 *m/z*) and the ^13^C_2_, ^15^N-acetamide internal standard (62 *m/z*). Peak areas of these ions were integrated and peak area ratios calculated (Chemstation software, RTE integrator, Agilent Technologies Inc, Sydney, Australia).

## Additional Information

**How to cite this article**: Liao, Y. *et al*. Developing a genetic manipulation system for the Antarctic archaeon, *Halorubrum lacusprofundi*: investigating acetamidase gene function. *Sci. Rep.* **6**, 34639; doi: 10.1038/srep34639 (2016).

## Supplementary Material

Supplementary Information

Supplementary Table S2

Supplementary Table S4

## Figures and Tables

**Table 1:** Strains and plasmids.

Strain or plasmid	Relevant properties	Reference or source
Plasmids		
pTA131	pBluescript II with *Bam*HI-*Xba*l I fragment from pGB70 containing pfdx-*pyrE2*; *bla* (Amp^R^)	31
pIDJL40	*gfp-*fusion expression vector derived from pTA962	39, 40
pJWID1	Shuttle vector derived from pTA962 with *pyrE2*, *hdrB*, *hmgA*, *bla*, SmRS-GFP and pHV2 origin of replication	This study
pTA131_Δ*amd3*	pTA131 with *Eco*RI-*Not*I fragment containing *hmgA* and *amd3* flanking regions for gene inactivation	This study
pJWID1_*amd3*	pJWID1 with *Nde*I-*Eco*RI fragment containing *amd3*	This study
Strains		
* E. coli* c2925	*dam, dcm* strain; used for preparing unmethylated plasmid DNA	New England Biolabs, C2925I
* Hfx. volcanii* DS2	Source of *hmgA* gene	33
* Hrr. lacusprofundi* ACAM34	Wild-type	7
* Hrr. lacusprofundi* Δ*amd3*	*Hrr. lacusprofundi* ACAM34 with *amd3* gene disruption	This study
