# Supplementary material for: Developing a genetic manipulation system for the Antarctic archaeon, Halorubrum lacusprofundi: investigating acetamidase gene function
Source: Sci Rep. 2016 Oct 6;6:34639. doi: 10.1038/srep34639 (PMC5052560; doi:10.1038/srep34639)
Supplement: Supplementary Information [file srep34639-s1.pdf]

Developing a genetic manipulation system for the Antarctic archaeon, *Halorubrum lacusprofundi*: investigating acetamidase gene function

Yan Liao, Timothy J. Williams, James C. Walsh, Muyan Ji, Anne Poljak, Paul M.G. Curmi, Iain G. Duggin and Ricardo Cavicchioli

Supplementary Information

Transformation protocol

Figures S1 – 11

Tables S1 – S4 (note Table S2 and S4 are provided separately as excel files)

Halorubrum lacusprofundi transformation protocol

The protocol is provided as a laboratory procedure explaining the steps performed each day of the experiment. Recipes for media and solutions are provided at the end of the procedure.

Plate out the *Hrr. lacusprofundi* ACAM34 strain on ADLVB solid medium and inoculate a single colony into 10 mL of ADLVB media. Incubate at 30°C (shaking, 120 rpm) for about 5 d (when the culture is turbid) and use 500 µL of this as an inoculum for a 50 mL culture in a 250 mL flask. Grow for approximately one more week (~ 7-10 d, shaking, 120 rpm) to provide a late exponential culture (quite turbid, and has a pink color, OD_{600} ~ 0.8).

Day 1

1. Ensure adequate quantities of transformation solutions are present (i.e. buffered and unbuffered spheroplasting solutions, spheroplast dilution solution and regeneration solution)
2. Leave PEG_{600} at RT to thaw overnight. Warm to 30°C if necessary.

Day 2

3. When the culture reaches OD_{600} ~0.8 (see above), pellet 10 mL by centrifuging at 5000 rpm for 20 min at 25°C. All subsequent steps are performed at room temperature unless otherwise stated.
4. Resuspend pellet gently in 2 mL of buffered spheroplasting solution. Transfer to 2 ml round-bottomed tube and pellet cells at 6000 rpm, 8 min at 25°C.
5. Very gently, resuspend in 200 µL of buffered spheroplasting solution (for each transformation reaction). Avoid generating air bubbles.
6. For each transformation, transfer 200 µL cells to a clean 2 ml round-bottomed tube. Add 20 µl of 0.5 M EDTA pH 8.0 onto the side of the tube, and invert to mix. Leave at RT for 10 min to form spheroplasts.
7. Meanwhile set up DNA samples in 30 µL total volumes:

    10 µl of unmethylated DNA (~1 µg, ultra-pure, prepared from *E. coli* c2925 strain) in DNase, RNase and protease-free water.

    15 µl of unbuffered spheroplasting solution.

    5 µl of 0.5 M EDTA pH 8.0.

8. Add the DNA samples (step 7) to the tubes containing the spheroplasts (step 6) to commence the transformation process.

9. After 5 min, add 250 µL (equal volume) of 60% PEG_{600} to each transformation tube. Add in same manner as EDTA (step 6), but shake the tube horizontally ~10 times to gently mix the solution. Leave at RT for 30 min.

10. Add 1.5 mL spheroplast dilution solution to each transformation tube, invert to mix and leave at RT for 2 min. Pellet using a microfuge at 12,000 rpm for 10 min at 25°C, and remove the supernatant.

11. Add 1 mL regeneration solution, resuspend the pellet gently until a homogeneous solution is obtained. Return to 30 °C for 4 h to regenerate cell walls (shaking, 120 rpm).

12. Pellet using a microfuge at 12,000 rpm for 10 min at 25°C. Remove supernatant and resuspend gently in 1 mL transformation dilution solution.

13. Plate 100 µL on ADLVB or ADLVB + antibiotic (selective) plates.

14. Incubate plates at 30 °C in sealed plastic bags for about 15 d. Place wet tissue into the bag to avoid media dehydration and salt crystallization. Check the plates every 3 d, and carefully remove condensation that has formed on the lid.

Artificial Deep Lake Vitamin succinate Broth (ADLVSB) media (1L)

NaCl	180.0 g,
$MgCl_2.6H_2O$	75.0 g,
Sodium succinate	10.0 g (Sigma),
$MgSO_4.7H_2O$	7.4 g,
KCl	7.4 g,
$CaCl_2.2H_2O$	1.0 g (Sigma),
Yeast Extract	1.0 g (Oxoid);

10 mL Vitamin solution containing per litre: Biotin 30.0 mg, Cyanocobalamin 20.0 mg, Thiamine- HCl 10.0 mg.

Adjust basal medium pH to 7.2 ± 0.2, and autoclave for 15 min at 15 psi and 121 °C. Filter sterilize vitamin solution and add to basal medium after autoclaving.

Pravastatin stock

5 mg ml^{-1} in pure water

30% BSW (buffered salt water) (1L)

NaCl	260 g,

$MgCl_2.6H_2O$	10 g,
$MgSO_4.7H_2O$	35 g,
KCl	7 g,
1 M Tris-HCl, pH7.4	20 mL

Dissolve completely in ~800 mL Milli-Q water, and then bring volume to 995 mL. Autoclave.
Add 5 mL sterile 1M $CaCl_2$.

10X YPC (100 mL)

Yeast extract	5 g
Oxoid peptone (not Difco Bacto)	1 g
Casamino acid	1 g

Dissolve in Milli-Q water and then bring pH to 7.4 with 2M KOH and the volume to 100 mL.
Use freshly or store in the fridge for a maximum of a few days.

Buffered Spheroplasting Solution

NaCl	5.844 g (1M)
KCl	0.2 g (27 mM)
1M Tris.HCl pH8.5	5 ml (50 mM)
Sucrose	15 g (15%)
dH_2O	to 100 mL

Filter sterilize

Unbuffered Spheroplasting Solution

NaCl	5.844 g (1M)
KCl	0.2 g (27 mM)
Sucrose	15 g (15%)
dH_2O	to 100 mL

Adjust to pH 7.5 (~ 10 µl 1 M NaOH). Filter sterilize

Spheroplast Dilution Solution

30% BSW	76.7 ml (23%)
Sucrose	15 g (15%)
0.5 M $CaCl_2$	0.75 ml (3.75 mM)
dH_2O	to 100 mL

Filter sterilize

Regeneration Solution

30% BSW	192 mL	(23%)
10X YPC	25 mL	(1X)
Sucrose	37.5 g	(15%)
dH_2O	to 250 mL	

Filter sterilize

Transformant Dillution Solution

30% SW	192 mL	(23%)
Sucrose	37.5 g	(15%)
dH_2O	to 250 mL	

Filter sterilize

Agar plates: After autoclaving the media, allow to cool to ~ 60 °C with stirring before adding components. Add the corresponding volume of pravastatin stock to make the final concentration required for each plate.

Figure S1. Growth of pJWID1 transformed and non-transformed *Hrr. lacusprofundi* in the presence of pravastatin. Transformed and non-transformed cells exhibited a large difference in their ability to grow on pravastatin containing solid media. Transformation was effective when selecting on plates using 2.5 µg mL^{-1} pravastatin. Growth assessments were semi-quantiative: ++++, > 100 colonies; +++, ≤ 100 colonies; ++, ≤ 50 colonies; +, ≤ 10 colonies; -, no visible growth; nd, not determined.

Figure S1

Type	Pravastatin concentration μg mL^{-1}									
	0	0.05	0.5	1	2.5	5	7.5	10	15	20
WTpJWID1	++++	nd	nd	++++	++++	+++	++	+	+	+
WT_control	++++	++++	+	+	-	-	-	-	-	-

WTpJWID1

WT_control

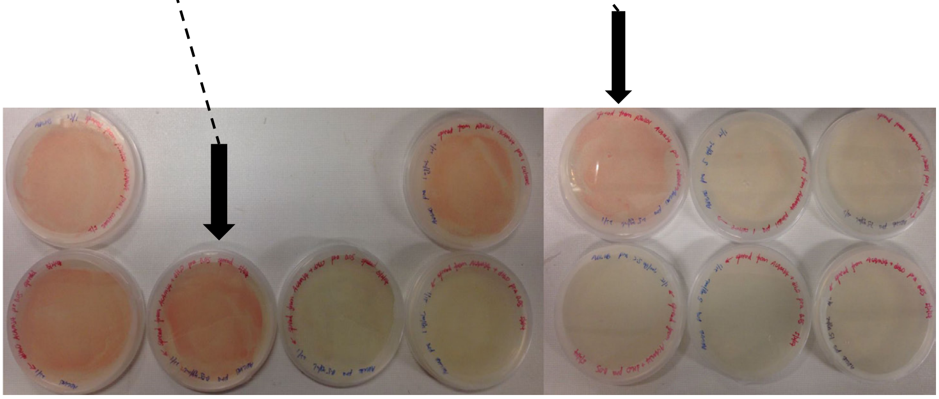

Pravastatin (μg mL^{-1}) 0 0.05 0.5 1 2.5 7.5 10

Figure S2. Effect of plasmid concentration on transformation efficiency of pJWID1 in *Hrr. lacusprofundi*. The transformation efficiency was calculated using different concentrations of plasmid, performing a dilution series and counting colonies only on plates containing 30 – 300 colonies, and expressing transformation efficiency as the number of transformants per µg of pJWID1 DNA. Pravastatin was used for selection of transformants at a concentration of 2.5 µg mL^{-1}.

Figure S2

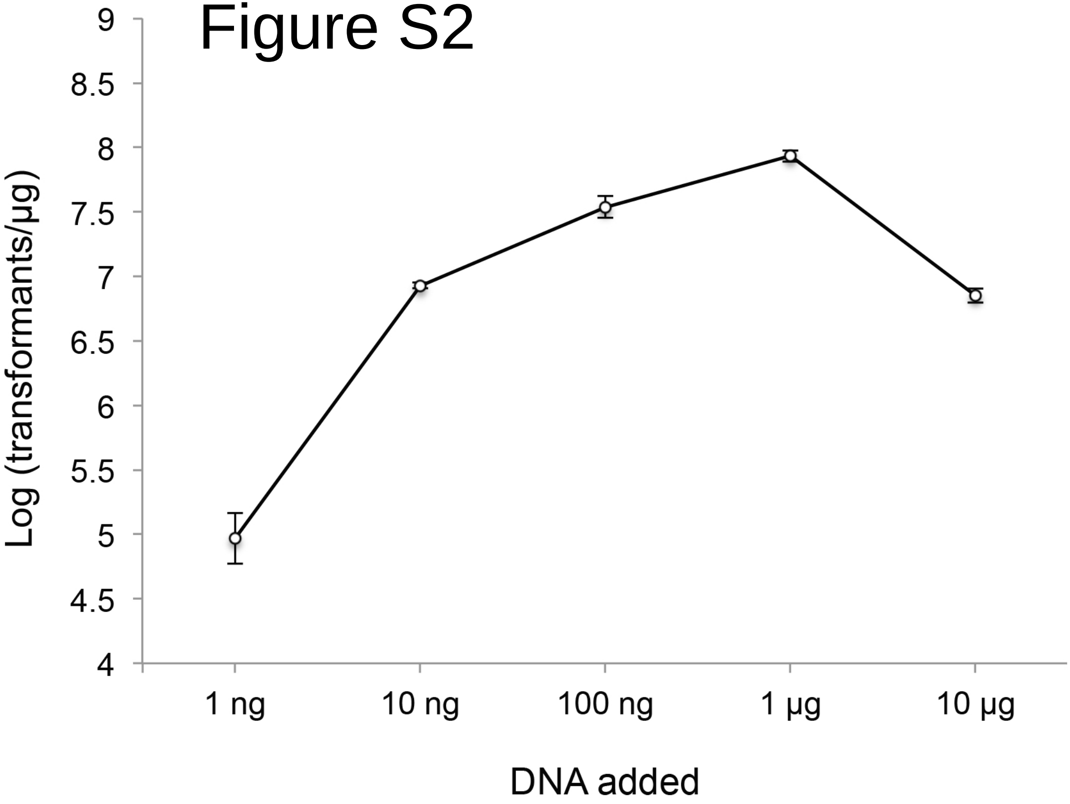

Log (transformants/µg)
9
8.5
8
7.5
7
6.5
6
5.5
5
4.5
4
1 ng
10 ng
100 ng
1 µg
10 µg
DNA added

Figure S3. Stability of pJWID1 in *Hrr. lacusprofundi*. A. The plasmid pJWID1 remained stable in transformed cells if selection pressure (2.5 µg mL^{-1}) was maintained. Plates show the pravastatin resistance (2.5 µg mL^{-1}) of colonies after cultures were grown in the presence of pravastatin (2.5 µg mL^{-1}). B. The plasmid pJWID1 was not maintained in *Hrr. lacusprofundi* without pravastatin selection. Plates show all colonies grown in media lacking pravastatin were unable to grow when subcultured on pravastatin (2.5 µg mL^{-1}) containing media. C. Wild-type *Hrr. lacusprofundi* cells were not resistant to pravastatin (2.5 µg mL^{-1}). As a control wild-type cells not harboring the plasmid only grew on plates that did not contain pravastatin.

Figure S3

A. with selection pressure (WTpJWID1)

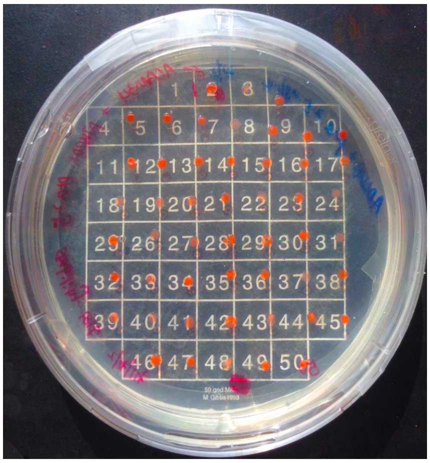

+ 2.5 μg mL^{-1} pravastatin

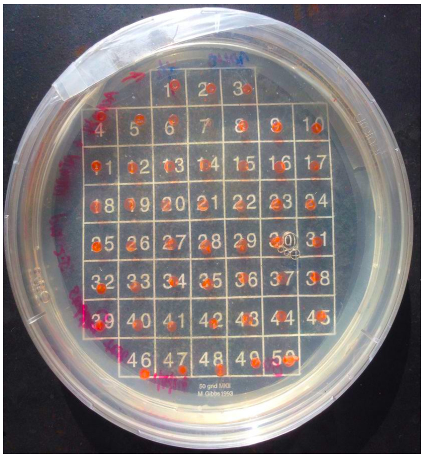

- pravastatin

B. without selection pressure (WTpJWID1)

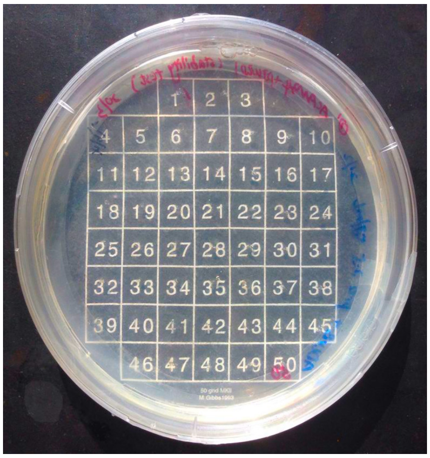

+ 2.5 μg mL^{-1} pravastatin

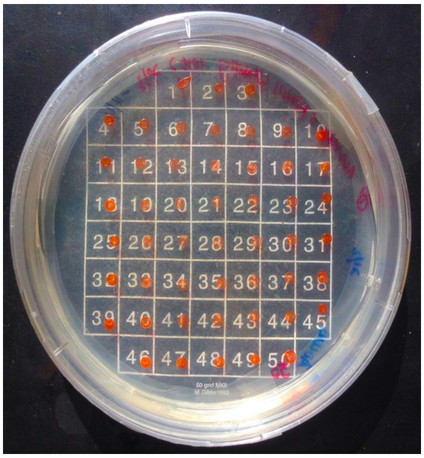

- pravastatin

C. negative control WT

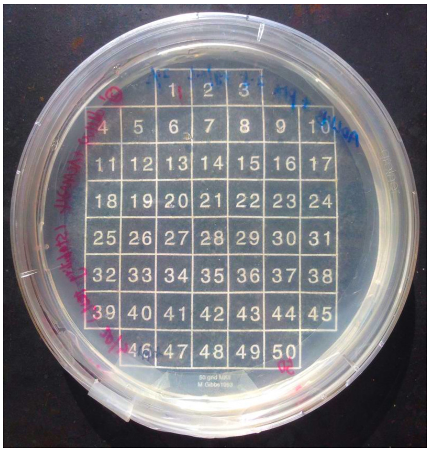

+ 2.5 μg mL^{-1} pravastatin

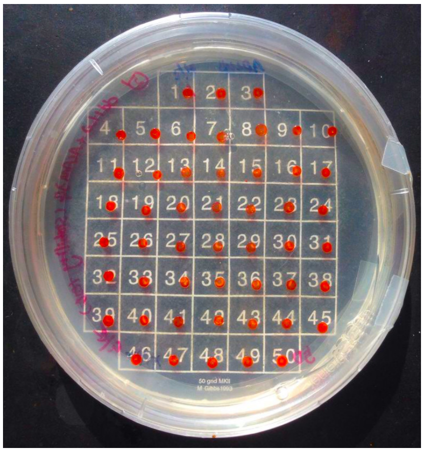

- pravastatin

Figure S4. Microscopy assessment of *Hrr. lacusprofundi* cells harboring plasmid pJWID1 following tryptophan induction of GFP expression. With tryptophan induction, particularly at the highest concentration tested (3 mM), fluorescence microscopy revealed fluorescing *Hrr. lacusprofundi* transformed cells. Bright field images provided for comparison. The bar represents 50 µm.

Figure S4

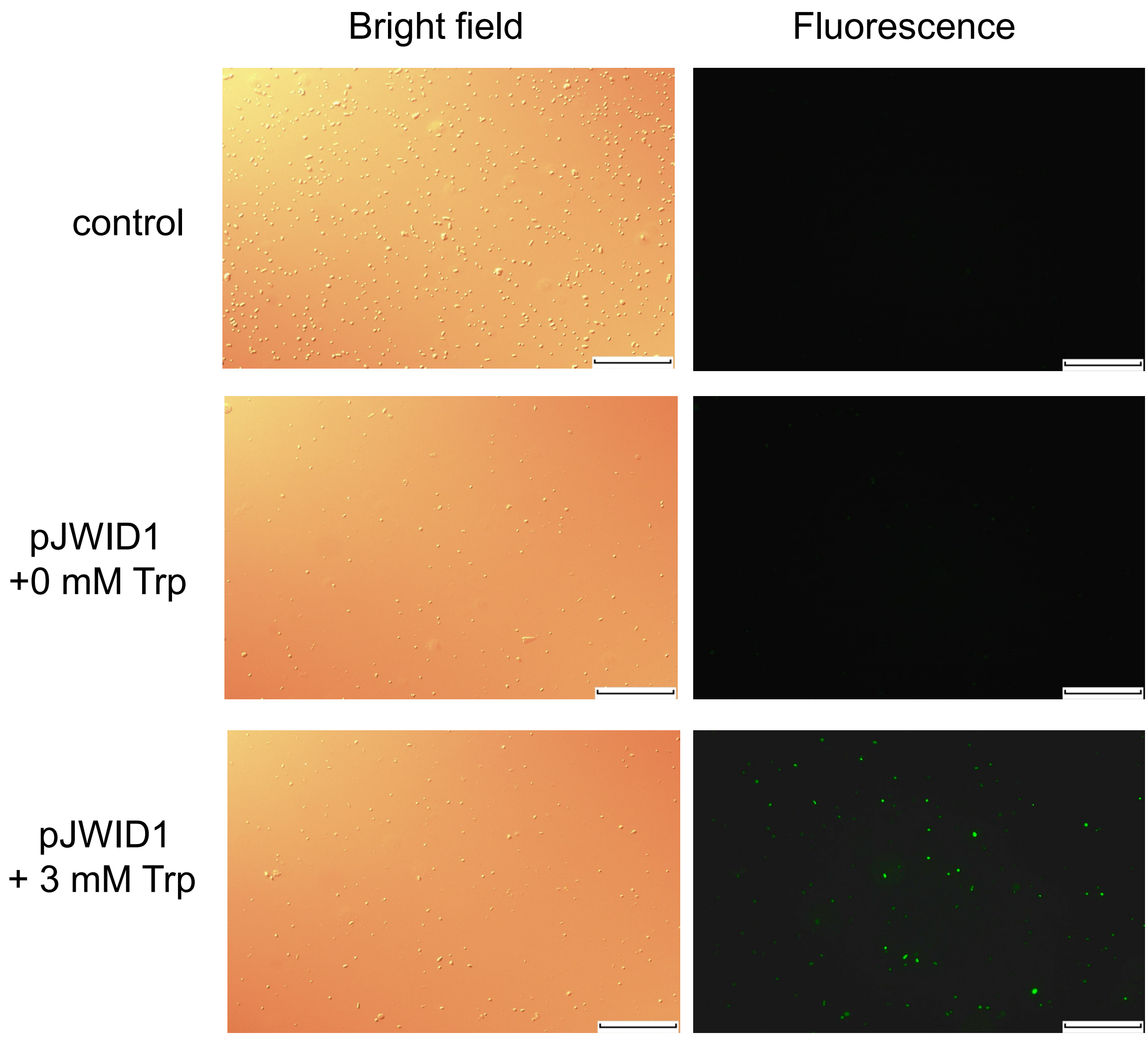
Bright field
Fluorescence
control
pJWID1
+0 mM Trp
pJWID1
+ 3 mM Trp

Figure S5. PCR assessment of recombination events in *Hrr. lacusprofundi* during the construction of the *amd3* gene disruption. Pravastatin resistant colonies amplified by PCR using primers: P1 (upper panel), P2 (lower panel). Lane M, 1 kb DNA ladder; Lane N, no DNA (negative control); Lane WT, untransformed *Hrr. lacusprofundi*; Lane C1-C10, randomly picked transformed colonies. Both C1 and C2 failed to show a band with P1 and P2 primers, consistent with them arising from a double recombination event (also see Fig. 3).

Figure S5

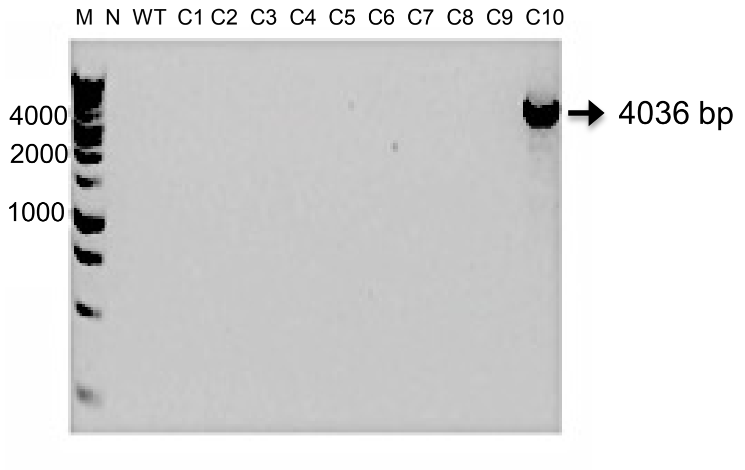
M N WT C1 C2 C3 C4 C5 C6 C7 C8 C9 C10
4000
2000
1000
4036 bp

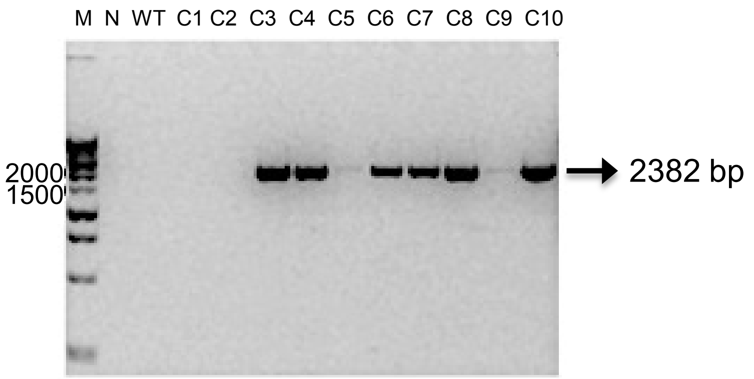
M N WT C1 C2 C3 C4 C5 C6 C7 C8 C9 C10
2000
1500
2382 bp

Figure S6. GFP expression in *Hrr. lacusprofundi* wild-type harboring pJWID1 or pJWID1_*amd3*. Fluorescence was measured after 3, 5 and 7 d: pJWID1, 0 mM tryptophan (horizontal stripes); pJWID1, 3 mM tryptophan (solid grey); pJWID1_*amd3*, 0 mM tryptophan (white); pJWID1_*amd3*, 3 mM tryptophan (black). The plasmids were maintained using 2.5 µg mL^{-1} pravastatin. Fluorescence from pJWID1_*amd3* was lower than pJWID1. Error bars represent standard error of three replicate cultures.

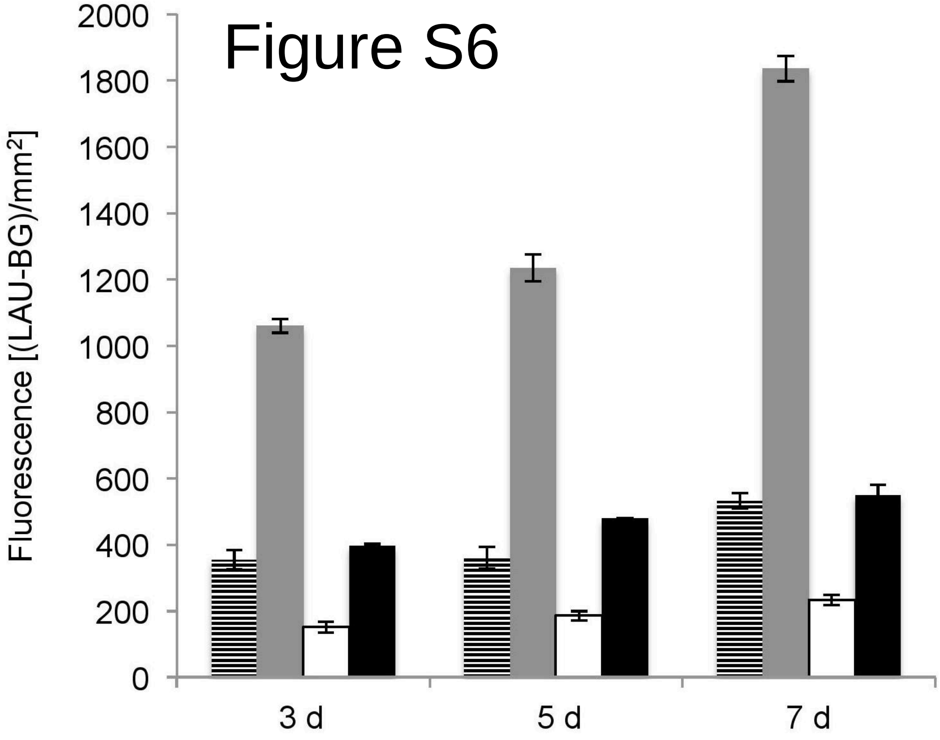
Figure S6
Fluorescence [(LAU-BG)/mm2]
2000
1800
1600
1400
1200
1000
800
600
400
200
0
3 d
5 d
7 d

Figure S7. Number of Amd/Fmd sequences within OUT0.9 clusters for *Archaea* and *Bacteria*. A. *Archaea*; B. *Bacteria*.

Figure S7

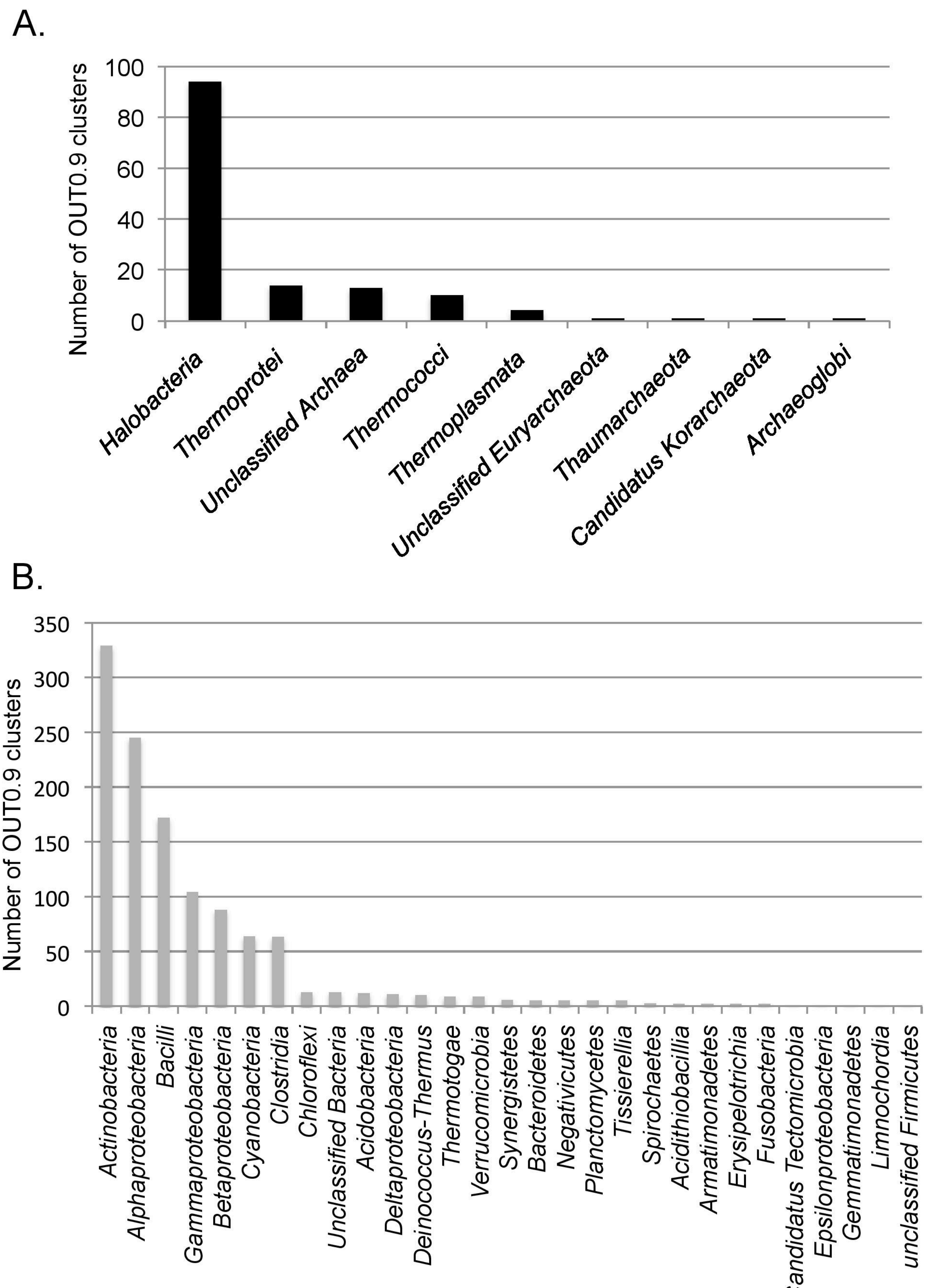
A.
Number of OUT0.9 clusters
100
80
60
40
20
0
Halobacteria
Thermoprotei
Unclassified Archaea
Thermococci
Thermoplasmata
Unclassified Euryarchaeota
Thaumarchaeota
Candidatus Korarchaeota
Archaeoglobi
B.
Number of OUT0.9 clusters
350
300
250
200
150
100
50
0
Actinobacteria
Alphaproteobacteria
Bacilli
Gammaproteobacteria
Betaproteobacteria
Cyanobacteria
Clostridia
Chloroflexi
Unclassified Bacteria
Acidobacteria
Deltaproteobacteria
Deinococcus-Thermus
Thermotogae
Verrucomicrobia
Synergistetes
Bacteroidetes
Negativicutes
Planctomycetes
Tissierellia
Spirochaetes
Acidithiobacillia
Armatimonadetes
Erysipelotrichia
Fusobacteria
Candidatus Tectomicrobia
Epsilonproteobacteria
Gemmatimonadetes
Limnochordia
unclassified Firmicutes

Figure S8. Maximum likelihood cladogram for Amd/Fmd sequences from *Archaea* and *Bacteria*. A total of 1323 clusters (OTU0.9) are shown. Clade 1, green lines; Clade II, blue lines; Clade III, red lines; Archaeal sequences within clades, black lines. The eight Amd/Fmd sequences from the Deep Lake haloarchaea are labelled: B9LQ06 (Amd1); B9LQH3 (Amd2); B9LRY7 (Amd3); G2MFV3 (Halar_0731); G2MKL2 (Halar_1208); G2MN64 (Halar_3390); halTADL_0419; halTADL_2650. The phylogenetic tree was tested by 1000 bootstraps, and the separation of three clades was supported by a bootstrap value of 0.996.

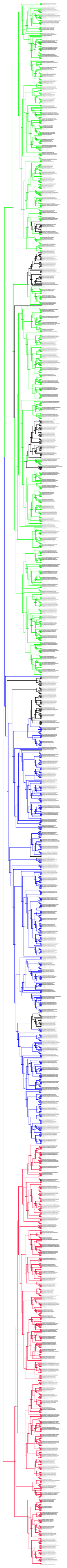

Figure S9. Maximum likelihood tree constructed from a subsample of equal numbers of Amd/Fmd clusters from *Archaea* and *Bacteria*. A total of 139 sequences were subsampled from bacterial OTU0.9 clusters to equal the number of archaeal clusters. The three clades root with a bacterial origin, similar to the full trees (Fig. 6 and Fig. S8). Clade 1, green lines; Clade II, blue lines; Clade III, red lines; Archaeal sequences within clades, black lines. Scale represents 1 amino acid variation per aligned position.

Figure S9

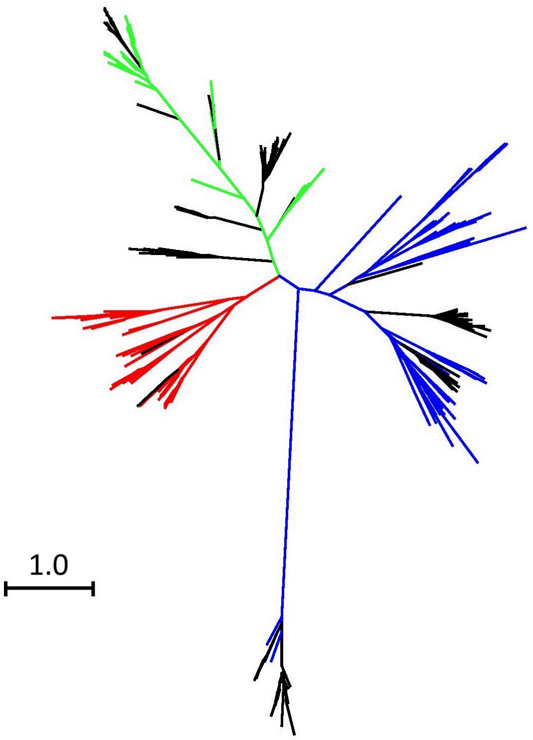

Figure S10. Number of Amd/Fmd sequences in closed genomes from *Archaea* and *Bacteria*. A. Average number of Amd/Fmd sequences within genomes that contain Amd/Fmd sequences. B. Average number of Amd/Fmd sequences within genomes of *Archaea* that contain Amd/Fmd sequences, with taxa shown at the class level. C. Average number of Amd/Fmd sequences within genomes of *Bacteria* that contain Amd/Fmd sequences, with taxa shown at the phylum/class level.

Figure S10

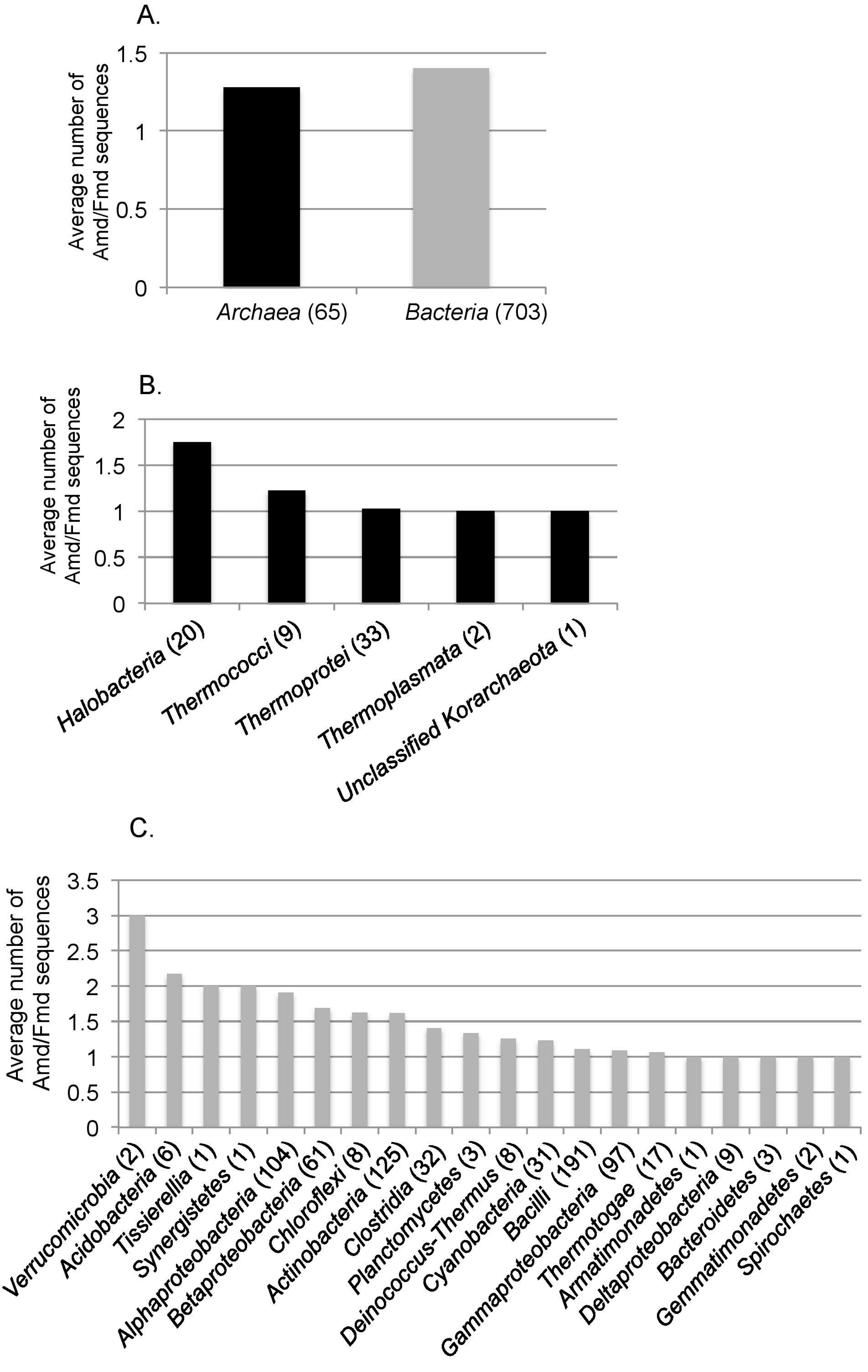

A.
Average number of Amd/Fmd sequences
1.5
1
0.5
0
Archaea (65)
Bacteria (703)
B.
Average number of Amd/Fmd sequences
2
1.5
1
0.5
0
Halobacteria (20)
Thermococci (9)
Thermoprotei (33)
Thermoplasmata (2)
Unclassified Korarchaeota (1)
C.
Average number of Amd/Fmd sequences
3.5
3
2.5
2
1.5
1
0.5
0
Verrucomicrobia (2)
Acidobacteria (6)
Tissierellia (1)
Synergistetes (1)
Alphaproteobacteria (104)
Betaproteobacteria (61)
Chloroflexi (8)
Actinobacteria (125)
Clostridia (32)
Planctomycetes (3)
Deinococcus-Thermus (8)
Cyanobacteria (31)
Bacilli (191)
Gammaproteobacteria (97)
Thermotogae (17)
Armatimonadetes (1)
Deltaproteobacteria (9)
Bacteroidetes (3)
Gemmatimonadetes (2)
Spirochaetes (1)

Figure S11. GC/MS analyses of acetamide. A. A five point acetamide standard series in the 0 – 20 µg mL^{-1} range fitted with a two point polynomial standard curve. B. GC/MS chromatogram of acetamide standards (*m/z* 59 ion), showing the typical elution time of ~28 min.

Figure S11

A.

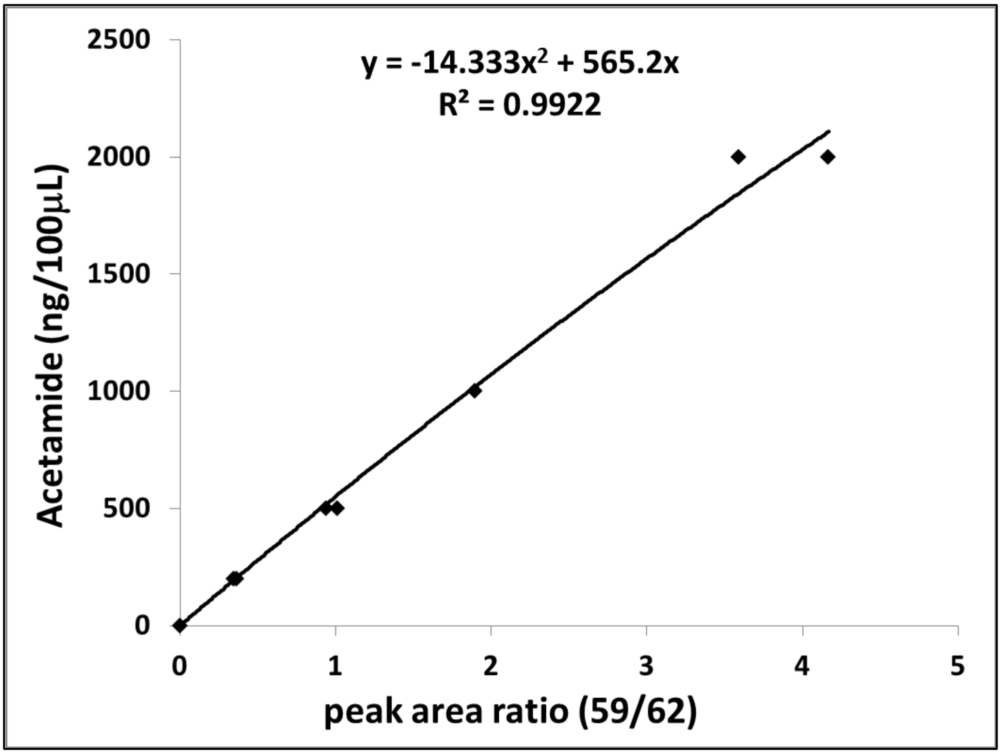

B.

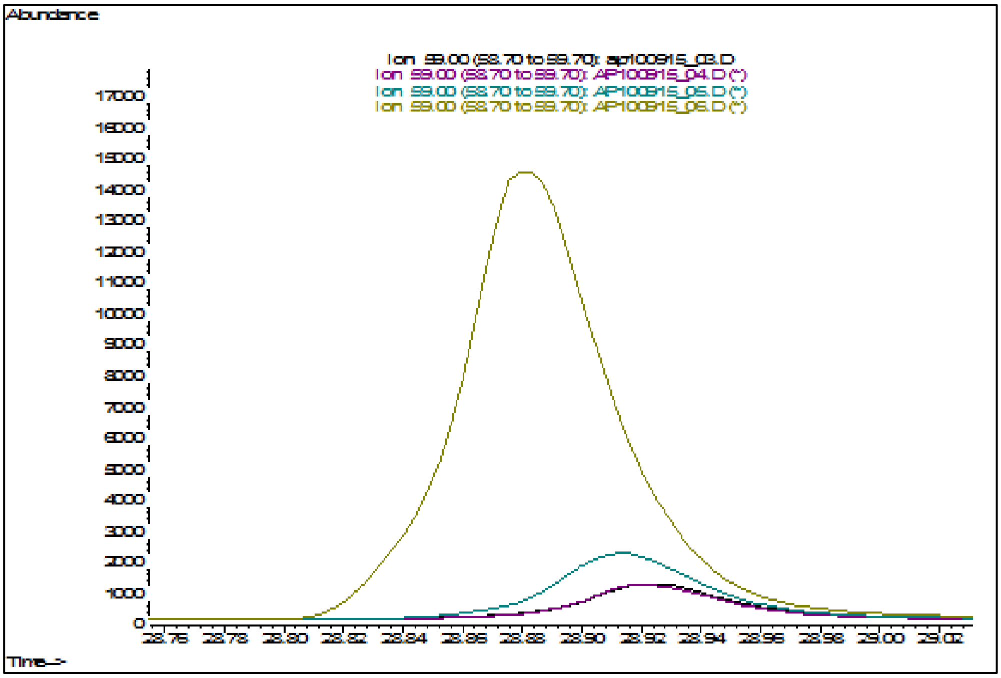

Table S1. Oligodeoxynucleotide primers used in this study.

Primers used for plasmid construction		
Primer name	Sequence	Description
pJ_For	GGCGGCCGTTGGAAGAACCGG	*hmgA* amplification for creating pJWID1, forward primer
pJ_For_M	GATACTGCAGAACGCTTTAAGGCCGGCGCGGG	*hmgA amplification* for creating pJWID1, mutated forward primer; promoter mutations sites underlined
pJ_Rev	GATACTGCAGTTACCGACCGAGTTCGGCGTGG	*hmgA amplification* for creating pJWID1, reverse primer
hmgA_For	GCAGGAAAGAACATGCAGAACG	*hmgA amplification* for creating pTA131_ *Δamd3*, forward primer
hmgA_Rev	CCATGCAGTTACCGACCGAGTT	*hmgA* amplification for creating pTA131_ *Δamd3*, reverse primer
Acet_down_For15_h	CATGTTCTTTCCTGCCCTCAAGAAGTTCGGCTACA	*amd3* deletion, 15 bp overlap with 5' end of *hmgA* (underlined)
Acet_down_Rev15	GCTTGATATCGAATTCGGATACGCCTGAATCGTGAG	*amd3* deletion, 15 bp overlap with *Eco*RI ends of pTA131 (underlined)
Acet_up_For15	ACCGCGGTGGCGGCCGCTCGTGGTCGTGGCTGTAGAGGTGG	*amd3* deletion, 15 bp overlap with *Not*I ends of pTA131 (underlined)
Acet_up_Rev15_h	TCGGTAACTGCATGGGGCGAGGTAGTGGACTTGCGTGAGG	*amd3* deletion, 15bp overlap with 3' end of *hmgA*
pJ_2285_FW	GACCTATTGCGCATATGATGCCAGAAGTCAAATTCGA	Complementation, 15 bp overlap with *Nde*I ends of pJWID1
pJ_2285_RV	CGGGCTGCAGGAATTCTCACTCAAGGGGATCGTC	Complementation, 15 bp overlap with *Eco*RI ends of pJWID1

Primer used in the PCR assessment for Hlac_2285 gene deletion				
Primer group-diagnostic purpose	Primer name	Primer location	Sequence	Product size
P1: single recombination, position 1	SR1_For	ColE1 of pTA131	ACTACGGCTACACTAGAAGGA	4036 bp
	SR1_Rev	*amd3*	CGGGAACCACGACATCAA	
P2: single recombination, position 2	SR2_For	*amd3*	TTGATGTCGTGGTTCCCG	2382 bp
	SR2_Rev	ColE1 of pTA131	TCCTTCTAGTGTAGCCGTAGT	
P3: double recombination, replacing *amd3* with *hmgA* (positive test)	DRP_For	Chromosome downstream of *amd3*	CAACCGTCGGATCAGTGAG	1193 bp
	DRP_Rev	*hmgA*	CGCTTCGAGTTCGTGGAG	
P4. double recombination, replacing *amd3* with *hmgA* (negative control)	DRP_For	Chromosome downstream of *amd3*	CAACCGTCGGATCAGTGAG	1423 bp
	SR1_Rev	*amd3*	CGGGAACCACGACATCAA	

Table S3. Taxonomic distribution of Amd/Fmd sequences in closed genomes of *Archaea* and *Bacteria*.

Taxonomic group	Number of genomes	Number of genomes containing Amd/Fmd sequences	Number of Amd/Fmd sequences	Average number of Amd/Fmd sequences in closed genomes containing Amd/Fmd sequences	Average number of Amd/Fmd sequences in all closed genomes
Archaea					
Euryarchaeota					
Halobacteria	30	20	35	1.75	1.17
Thermococci	19	9	11	1.22	0.58
Thermoplasmata	7	2	2	1	0.29
Archaeoglobi	7	0	0	-	0
Methanobacteria	13	0	0	-	0
Methanococci	15	0	0	-	0
Methanomicrobia	47	0	0	-	0
Methanopyri	1	0	0	-	0
unclassified	3	0	0	-	0
Korarchaeota					
unclassified	1	1	1	1	1
Crenarchaeota					
Thermoprotei	58	33	34	1.03	0.59
Thaumarchaeota					
unclassified	12	0	0	-	0
Nanoarchaeota					
unclassified	1	0	0	-	0
**Unclassified *Archaea***	1	0	0	-	0
**Total**	215	65	83	1.28	0.39
Bacteria					
Acidobacteria	8	6	13	2.17	1.63
Actinobacteria	425	125	202	1.66	0.49
Aquificae	14	0	0	-	0
Armatimonadetes	2	1	1	1	0.5
Bacteroidetes	138	3	3	1	0.02
Caldiserica	1	0	0	-	0
Candidatus Saccharibacteria	2	0	0	-	0
Chlamydiae	108	0	0	-	0
Chlorobi	11	0	0	-	0
Chloroflexi	25	8	13	1.7	0.52
Cloacimonetes	1	0	0	-	0
Cyanobacteria	91	31	38	1.2	0.42
Deferribacteres	4	0	0	-	0

Deinococcus-Thermus	21	8	10	1.25	0.48
Dictyoglomi	2	0	0	-	0
Elusimicrobia	3	0	0	-	0
Fibrobacteres	1	0	0	-	0
Bacilli	703	191	210	1.10	0.30
Clostridia	178	32	45	1.41	0.25
Tissierellia	3	1	2	2	0.67
Negativicutes	8	0	0	-	0
Erysipelotrichia	1	0	0	-	0
Fusobacteria	20	0	0	-	0
Gemmatimonadetes	2	2	2	1	1
Kryptonia	2	0	0	-	0
Nitrospirae	6	0	0	-	0
Planctomycetes	7	3	4	1.33	0.57
Alphaproteobacteria	350	104	198	1.90	0.57
Betaproteobacteria	289	61	103	1.69	0.36
Deltaproteobacteria	70	9	9	1	0.13
Gammaproteobacteria	970	97	105	1.08	0.11
Epsilonproteobacteria	155	0	0	-	0
Spirochaetes	82	1	1	1	0.01
Synergistetes	5	1	2	2	0.4
Tenericutes	129	0	0	-	0
Thermodesulfobactera	3	0	0	-	0
Thermotogae	23	17	18	1.06	0.78
Verrucomicrobia	7	2	6	3	0.86
Unclassified *Bacteria*	2	0	0	-	0
**Total**	3872	703	985	1.40	0.25
